# Cell-free secretome of CD56^bright^CD16^bright^ directly reprogrammed NK cells enhances wound healing via CCL3/4/5-CCR5 signaling

**DOI:** 10.7150/thno.120219

**Published:** 2026-01-01

**Authors:** Jae Yun Kim, Han-Seop Kim, Binna Seol, Ji Eun Choi, Ji-Young Lee, Yee Sook Cho

**Affiliations:** 1 Stem Cell Research Laboratory, Immunotherapy Research Center, Korea Research Institute of Bioscience and Biotechnology (KRIBB), 125 Gwahak-ro, Yuseong-gu, Daejeon 34141, Republic of Korea.; 2 Department of Bioscience, KRIBB School, University of Science & Technology, 113 Gwahak-ro, Yuseong-gu, Daejeon 34113, Republic of Korea.

**Keywords:** NK cells, secretome, wound healing, angiogenesis, regenerative medicine

## Abstract

**Rationale:** Natural killer (NK) cells are emerging as a promising source of immunomodulatory secretomes with regenerative potential. However, heterogeneity in primary NK cell populations limits the reproducibility of NK-derived cell-free therapies. To address this, we developed directly reprogrammed NK (drNK) cells with a stable CD56^bright^CD16^bright^ phenotype and investigated the therapeutic potential of their conditioned medium (drNK-CM) in wound healing, focusing on underlying molecular mechanisms such as chemokine signaling and angiogenesis.

**Methods:** drNK cells were generated by transcription factor-mediated reprogramming (OCT4, SOX2, KLF4, MYC) and characterized via flow cytometry and RNA-seq. The secretome profile of drNK-CM was evaluated using proteomic analysis. Human epidermal keratinocytes (HEKs), dermal fibroblasts (HDFs), and endothelial cells (HUVECs) were treated with drNK-CM to assess proliferation, migration, and extracellular matrix (ECM) remodeling. Chemokine receptor involvement was evaluated using CCR1, CCR3, and CCR5 antagonists. *In vivo* efficacy was tested in mouse excisional wound models, with histological and immunofluorescence evaluation of angiogenesis, re-epithelialization, and collagen deposition.

**Results:** drNK-CM significantly promoted proliferation and migration of HEKs, HDFs, and HUVECs, accompanied by enhanced expression of Type I/III collagen, VEGF, and MMPs. Transcriptomic profiling revealed that drNKs uniquely upregulated genes associated with ECM remodeling, chemokine signaling (CCL3/4/5), and angiogenesis. Notably, CCR5 inhibition by maraviroc abrogated drNK-CM-induced cell migration and delayed wound closure *in vivo*, highlighting the central role of the CCL3/4/5-CCR5 axis. Furthermore, drNK-CM activated AKT and ERK pathways and promoted anti-inflammatory macrophage polarization. *In vivo* application of drNK-CM accelerated wound closure, improved neovascularization, and supported organized tissue regeneration compared to controls.

**Conclusion:** This study demonstrates that drNK-CM enhances wound healing through coordinated actions on epithelial, stromal, and endothelial compartments. The reparative effects are primarily mediated via the CCL3/4/5-CCR5 signaling axis and pro-angiogenic cascades. Given their consistent phenotype and reproducible secretome, drNKs represent a scalable and safe source for cell-free regenerative therapeutics.

## Introduction

Natural killer (NK) cells are key effectors of the innate immune system, best known for their ability to recognize and eliminate abnormal or stressed cells through perforin- and granzyme-mediated cytotoxicity 1. Beyond this classical role in immune surveillance, NK cells exert potent immunomodulatory and regenerative effects by releasing a broad repertoire of bioactive molecules that orchestrate inflammation, extracellular matrix (ECM) remodeling, and angiogenesis 2-4. The NK cell secretome, composed of cytokines, chemokines, and growth factors, facilitates immune cell recruitment, modulates local inflammatory responses, and supports vascularization, thereby positioning NK cells as an emerging source for regenerative medicine.

Wound healing is a dynamic and tightly regulated process encompassing sequential and overlapping phases of inflammation, matrix deposition and remodeling, re-epithelialization, and neovascularization 5. When dysregulated, as in chronic wounds, this process is impaired by persistent inflammation, defective fibroblast migration, and insufficient angiogenesis, resulting in a major clinical burden 5. Mesenchymal stem cells (MSCs) and related cell-based therapies have shown promise in regenerative medicine, but challenges including tumorigenicity, immune compatibility, and manufacturing scalability continue to limit their translation 6-8. These limitations have accelerated interest in cell-free strategies that harness paracrine factors as therapeutics.

Accumulating evidence indicates that NK cell-derived factors directly promote fibroblast proliferation, collagen synthesis, and vascular remodeling, processes essential for tissue repair 9-12. NK cell-conditioned medium (NK-CM) has been reported to enhance keratinocyte migration and fibroblast proliferation, further supporting its potential in regenerative applications 2, 3, 13-19. Peripheral blood-derived NK-CM (pNK-CM) is enriched in pro-survival, angiogenic, anti-inflammatory mediators 12, 20, 21, reinforcing the therapeutic promise of NK cell secretomes as cell-free regenerative agents.

Importantly, NK cells are heterogeneous and highly plastic, with their phenotype and function shaped by environmental cues such as TGF-β, hypoxia, and metabolic stress 1, 22-24. While circulating subsets, CD56^dim^CD16^bright^ NK cells, which comprise > 90% of peripheral NK cells, are specialized for cytotoxicity, CD56^bright^CD16^dim^ NK cells (< 10% of NKs) are distinguished by high cytokine production 25-27. Beyond these, rare tissue-resident CD56^bright^CD16^bright^ NK cells uniquely combine potent cytokine secretion with reparative capacity 28, 29, but their scarcity limits translational use.

Recent advances in cellular reprogramming offer a strategy to overcome this limitation. Direct reprogramming with OCT4, SOX2, KLF4, and MYC (OSKM) enables the scalable generation of directly reprogrammed NK cells (drNKs) that consistently adopt a CD56^bright^CD16^bright^ phenotype 30. This reprogramming strategy addresses the scarcity of naturally occurring CD56^bright^CD16^bright^ NK cells by enabling scalable generation of drNKs from accessible somatic sources. Defining the regenerative functions of drNK-derived secretomes is essential, as they may provide a cell-free therapeutic platform that combines the reparative potential of rare NK subsets with the practicality and safety required for clinical translation.

Here, we investigate the regenerative activity of drNK-CM in both *in vitro* and *in vivo* models of skin wound healing. By directly comparing drNK-CM with pNK-CM, we sought to elucidate the mechanisms underlying the therapeutic efficacy of drNK-secreted factors. This study establishes drNK-CM as a novel, scalable, and cell-free therapeutic candidate for chronic wound repair and tissue regeneration.

## Materials and Methods

### Animal experiments

All animal procedures were conducted in accordance with institutional guidelines and approved by the Institutional Animal Care and Use Committee of the Korea Research Institute of Bioscience and Biotechnology (KRIBB) (Approval No. KRIBB-AEC-24105). Male C57BL/6J mice (8-10 weeks old; Dae Han BioLink Co., Ltd., Chungbuk, South Korea) were housed under specific pathogen-free (SPF) conditions with ad libitum access to food and water and maintained on a 12 h light/dark cycle. Mice were acclimated for at least 1 week prior to experimentation.

### Isolation and culture of peripheral blood NK cells

Peripheral blood mononuclear cells (PBMCs) were isolated from whole blood obtained from healthy donors (Korea Red Cross blood center), following approval from the Institutional Review Board of KRIBB (IRB No. P01-201812-31-010). PBMCs were isolated using Ficoll-Paque™ PREMIUM (GE Healthcare Life Sciences, Uppsala, Sweden) density gradient centrifugation and cultured in RPMI 1640 medium (Thermo Fisher Scientific) supplemented with 10% fetal bovine serum (FBS).

To isolate primary NK cells (pNKs), PBMCs (1 × 10⁷) were subjected to negative selection using a MACS NK cell isolation kit (Miltenyi Biotec, Bergisch Gladbach, Germany), according to the manufacturer's protocol. Biotin-conjugated antibodies and magnetic microbeads were used to deplete non-NK cells, and the NK cell-enriched fraction was collected from the flow-through. Purified pNKs were maintained in RPMI 1640 medium supplemented with 10% FBS, 1% penicillin/streptomycin (P/S), and 200 IU/mL recombinant human IL-2 (PeproTech). Culture medium was replaced every 2-3 days.

### Generation of directly reprogrammed NK cells

Directly reprogrammed NK cells (drNKs) were generated from PBMCs of four independent healthy donors (n = 4) by Sendai viral-mediated ectopic expression of OCT4, SOX2, KLF4, and MYC (OSKM) as previously described 30. PBMCs were first pre-cultured for 4 days in starting cell medium (SCM; StemPro-34 SFM supplemented with 2.5% nutrient supplement, 1% P/S, 2 mM GlutaMAX I, 20 ng/mL IL-3, 20 ng/mL IL-6, 100 ng/mL SCF, and 100 ng/mL FLT-3). Pre-cultured cells (3 × 10⁵) were then transduced with the non-integrating, temperature-sensitive Sendai virus (CytoTune 2.0, Thermo Fisher) carrying human OCT4 (O), SOX2 (S), KLF4 (K), and c-MYC (M) at defined multiplicities of infection (OSK:K:M = 5:3:5) for 24 h (day 0). On the following day, cells were transferred to reprogramming induction medium (RIM; 10% FBS, 1% P/S, 20 ng/mL IL-3, 20 ng/mL IL-6, 25 ng/mL SCF, 25 ng/mL FLT-3, and 25 ng/mL TPO in StemSpan SFEM II) supplemented with 5 µM CHIR99021 for 5 days. Cells were subsequently maintained in reprogramming maturation medium (RMM; 10% FBS, 1% P/S, 200 IU IL-2, 20 ng/mL IL-7, 20 ng/mL IL-15, 25 ng/mL SCF, and 25 ng/mL FLT-3 in StemSpan SFEM II) supplemented with 2 µM SR1 for 18 days (to day 24). Following reprogramming, emergent drNKs were maintained in feeder-free conditions using NK medium (RPMI 1640 with 10% FBS, 200 IU IL-2, and 10 ng/mL IL-15), with medium changes every 2-3 days. No feeder cells were required for expansion, and cultures could be stably maintained for > 40 days. Phenotypic stability of the CD56^bright^CD16^bright^ population was confirmed by flow cytometry.

### Preparation of conditioned medium

Conditioned media were collected from cultures of either pNKs (pNK-CM) or drNKs (drNK-CM). Freshly prepared pNKs and drNKs were seeded at a density of 1.0 × 10⁶ cells/mL in T75 flasks with 10 mL of culture medium and incubated for 24 h at 37 °C in a humidified atmosphere with 5% CO₂. Supernatants were harvested and centrifuged at 1,500 rpm for 5 min at 4 °C to remove residual cells and debris. The clarified media were filtered through 0.22 μm syringe filters (Millipore, Burlington, MA, USA) and concentrated using Amicon Ultra-15 centrifugal filter units (10 kDa cutoff; Millipore) at 4,000 × g for 30 min at 4 °C. drNK-CM was prepared separately from each of the four donors (n = 4). Cytokine and growth factor profiles were analysed using multiplex cytokine arrays and ELISA. The concentrated CM (CCM) was either used immediately or stored at -80 °C until further experiments.

### Flow cytometric analysis

Surface marker expression on NK cells was assessed by flow cytometry. Freshly prepared pNKs and drNKs were washed with phosphate-buffered saline (PBS) and resuspended in FACS buffer (PBS supplemented with 1 mM EDTA and 2% FBS) at a concentration of 1 × 10⁶ cells per 100 µL. Cells were stained for 30 min at 4 °C in the dark with APC-conjugated anti-human CD56 (Miltenyi Biotec, Cat# 130-113-598) and PE-conjugated anti-human CD16 (Miltenyi Biotec, Cat# 130-113-395). Corresponding isotype control antibodies were used for proper gating. After staining, cells were washed with FACS buffer, resuspended in 500 μL of PBS, and analyzed using a BD Accuri C6 flow cytometer (BD Biosciences, San Jose, CA, USA). Flow cytometry data were analyzed with FlowJo software (Tree Star, Ashland, OR, USA).

### Cell culture of dermal, epidermal, and endothelial Cells

Primary human dermal fibroblasts (HDFs; PCS-201-012™), epidermal keratinocytes (HEKs; PCS-200-011™), and human umbilical vein endothelial cells (HUVECs; PCS-100-010™) were obtained from the American Type Culture Collection (ATCC, Manassas, VA, USA). HDFs were cultured in Dulbecco's Modified Eagle Medium (DMEM; Invitrogen, Carlsbad, CA, USA) supplemented with 10% FBS and 1% P/S). HEKs were maintained in EpiLife® medium (M-EPI-500-CA; Thermo Fisher Scientific, Waltham, MA, USA) supplemented with human keratinocyte growth supplement (S-001-K; Thermo Fisher Scientific) and 1% P/S. HUVECs were cultured in Endothelial Cell Growth Medium-2 (EGM-2) BulletKit (CC-3162; Lonza, Walkersville, MD, USA). All cell types were grown as monolayers in T-25 culture flasks at 37 °C in a humidified atmosphere with 5% CO₂. Culture media were changed every other day, and cells were subcultured once or twice weekly upon reaching 80-90% confluence.

### Secretome profiling using cytokine array

The secretome composition of NK-CM was evaluated using the Proteome Profiler Human Cytokine Array Kit (ARY005B; R&D Systems, Minneapolis, MN, USA), following the manufacturer's instructions. CM was collected independently from four drNK cultures (n = 4) and matched primary NK controls. Briefly, array membranes were blocked with blocking buffer for 1 h at room temperature (RT), then incubated with 500 μL of NK-CM pre-mixed with a biotinylated antibody cocktail for 1 h at RT. The mixture was subsequently transferred to the blocked membranes and incubated overnight at 4 °C on a rocking platform. Following extensive washes, membranes were incubated with streptavidin-conjugated horseradish peroxidase (HRP) for 30 min at RT and then with chemiluminescent detection reagents for 1 min. Signal intensities were visualized using a chemiluminescence imaging system (Amersham Imager 680; GE Healthcare, Chicago, IL, USA). Densitometric analysis was performed using ImageJ software (NIH, Bethesda, MD, USA), and cytokine levels were normalized to internal positive control spots on the array.

### Quantification of secreted chemokines by ELISA

Levels of chemokines CCL3, CCL4, and CCL5 in NK-CM were quantified using commercial ELISA kits according to the manufacturers' instructions: Human CCL3/MIP-1α Quantikine ELISA Kit (DMA00; R&D Systems), Human CCL4/MIP-1β Quantikine ELISA Kit (DMB00; R&D Systems), Human CCL5/RANTES ELISA Kit (DRN00B; R&D Systems), and Granzyme B using the Human Granzyme B ELISA Kit (ab46142; Abcam). Measurements were performed on CM prepared separately from four drNK cultures (n = 4). Briefly, 100 μL of each standard or CM sample was added to 96-well plates pre-coated with capture antibodies and incubated for 2 h at RT. After washing, wells were incubated with HRP-conjugated detection antibody for 1 h, followed by additional washes and incubation with substrate solution for 30 min in the dark. The reaction was terminated by adding 50 μL of stop solution, and absorbance was measured at 450 nm using a microplate reader (SpectraMax i3; Molecular Devices, San Jose, CA, USA), with 540 nm as reference wavelength.

### Gene expression analysis by quantitative real-time PCR

Total RNA was isolated from cultured cells or tissue samples using the RNeasy Mini Kit (Qiagen, Hilden, Germany), following the manufacturer's protocol. RNA concentration and purity were assessed by spectrophotometry, and 2 μg of total RNA was reverse-transcribed into complementary DNA (cDNA) using the Script™ cDNA Synthesis Kit (Bio-rad, Hercules, CA, USA). Quantitative real-time PCR (qRT-PCR) was carried out using Fast SYBR™ Green Master Mix (Applied Biosystems, Foster City, CA, USA) on an ABI 7500 Fast Real-Time PCR System. Each 20 μL reaction consisted of 10 μL SYBR Green Master Mix, 1 μL of each forward and reverse primer (10 μM), 1 μL of cDNA template, and 8 μL of nuclease-free water. Amplification was performed in triplicate for each sample. GAPDH was used as the endogenous reference gene, and relative gene expression was calculated using the 2^-ΔΔCT^ method. Primer sequences for target and reference genes are listed in Table S1.

### Western blotting for protein expression analysis

For protein expression analysis, cells or wound tissue samples were lysed using RIPA buffer (25 mM Tris-HCl pH 7.6, 150 mM NaCl, 1% NP-40, 1% sodium deoxycholate, and 0.1% SDS) supplemented with protease (P3100) and phosphatase (P3200) inhibitor cocktails (GenDEPOT, Barker, TX, USA). Homogenization of wound tissues was performed using a Precellys 24 tissue homogenizer (Bertin Instruments, Montigny-le-Bretonneux, France). Equal amounts of protein (20 μg) were resolved by SDS-PAGE and transferred onto polyvinylidene difluoride (PVDF) membranes (Millipore). The membranes were blocked with 5% skim milk in TBS-T for 1 h at RT, followed by overnight incubation at 4 °C with primary antibodies. After washing, membranes were incubated with appropriate HRP-conjugated secondary antibodies. Detection was performed using the SuperSignal West Femto Chemiluminescent Substrate (Thermo Fisher Scientific), and chemiluminescent signals were captured with the ChemiDoc Imaging System (Bio-Rad, Hercules, CA, USA). Band intensities were quantified using ImageJ software and normalized to GAPDH or β-actin as internal loading controls. A full list of primary and secondary antibodies used is provided in Table S2.

### Assessment of cell proliferation and viability using MTT assay

To assess the effects of NK-CM and chemokine receptor inhibitors on cell proliferation and viability, HDFs, HEKs, and HUVECs were seeded at a density of 1.5 × 10⁴ cells per well in 96-well plates and allowed to adhere overnight. Cells were then treated with increasing concentrations (0%, 2.5%, 5%, 10% or 20%) of pNK-CM or drNK-CM for 48 h. In separate experiments, cells were co-treated with chemokine receptor antagonists including J113863, SB328437 (Tocris Bioscience, Bristol, UK), or maraviroc (MVC; Sigma-Aldrich, St. Louis, MO, USA) at concentrations of 0, 5, 10, 20, or 50 μM. Cell proliferation and viability were measured using the MTT-based Cell Proliferation Kit I (Roche, Cat# 11465007001, Mannheim, Germany) according to the manufacturer's protocol. After treatment, 10 μL of MTT reagent was added to each well and incubated for 4 h at 37 °C. The resulting formazan crystals were solubilized using the provided solubilization buffer, and absorbance was measured at 570 nm with a reference wavelength of 630 nm using a SpectraMax i3 microplate reader (Molecular Devices). Proliferation and viability were quantified using the following formulas: Proliferation rate (%) = (OD_treated_ - OD_day 0_) / (OD_Control_ - OD_day 0_) × 100; Relative cell viability (%) = (OD_treated_ / OD_Control_) × 100.

### *In vitro* wound healing assay using scratch method

A scratch assay was conducted to evaluate the effect of NK cell-conditioned media on cell migration during wound healing. HDFs, HEKs, and HUVECs were seeded at a density of 2 × 10⁵ cells *per* well in 24-well plates and cultured to full confluence. A uniform linear wound was introduced using the SPLScar™ Scratcher (SPL Life Sciences, Pocheon, South Korea), followed by gentle PBS washing to remove detached cells. Cells were then incubated in serum-free medium supplemented with the indicated concentrations of pNK-CM or drNK-CM, in the presence or absence of neutralizing antibodies against CCL3 (10 ng/mL; R&D Systems, Minneapolis, MN, USA), CCL4 (10 ng/mL; R&D Systems), or CCL5 (10 ng/mL; R&D Systems), as well as MVC (10 μM), J113863 (10 μM), or SB328437 (10 μM). Images of the scratch area were captured at 0 h and 48 h using a TE2000-E phase-contrast inverted microscope (Nikon, Tokyo, Japan), ensuring consistent imaging of predefined coordinates. After imaging, cells were fixed with 4% paraformaldehyde and stained with 0.5% crystal violet (Sigma-Aldrich) to visualize migration. Quantification of wound closure was performed using ImageJ software. The percentage of wound closure was calculated as follows: Wound closure (%) = (Area at 0 h - Area at 48 h) / Area at 0 h × 100.

### Immunohistochemistry analysis of collagen and endothelial/inflammatory markers

Immunohistochemistry (IHC) was performed to evaluate the expression of type I collagen (COL1A1), chemokine receptors (CCR1, CCR3, CCR5), and the endothelial marker CD31 in both cultured cells and wound tissues. For *in vitro* analysis, HDFs and HEKs were cultured on glass coverslips in 24-well plates to approximately 80% confluence. Cells were fixed with 4% paraformaldehyde (PFA; Sigma-Aldrich) for 15 min at RT, permeabilized with 0.1% Triton X-100 (Sigma-Aldrich) for 10 min, and washed with PBS. After blocking with 5% bovine serum albumin (BSA) for 1 h at RT, cells were incubated overnight at 4 °C with primary antibodies against COL1A1, CCR1, CCR3, CCR5, or CD31. The following day, cells were washed and incubated with Alexa Fluor 488-conjugated secondary antibodies for 1 h at RT in the dark. Nuclei were counterstained with 4′,6-diamidino-2-phenylindole (DAPI; Sigma-Aldrich) for 5 min. Fluorescence images were obtained using an Axio Vert.A1 fluorescence microscope (Carl Zeiss, Oberkochen, Germany). Marker-specific fluorescence intensity was quantified using ImageJ software and normalized to DAPI signal.

For tissue-level IHC, wound-healed skin samples were harvested 10 days post-surgery, fixed in 10% neutral buffered formalin (NBF; Sigma-Aldrich) overnight at 4 °C, and embedded in paraffin. Tissue sections (10 μm) were prepared using a rotary microtome (Leica RM2235; Leica Biosystems, Wetzlar, Germany) and mounted on glass slides. Antigen retrieval was performed by heating sections in 0.01 M citrate buffer (pH 6.0) at 95 °C for 20 min after deparaffinization and rehydration. Endogenous peroxidase activity was quenched with 3% hydrogen peroxide (H₂O₂) for 10 min. Sections were blocked with 5% BSA and incubated overnight at 4 °C with primary antibodies against CCR5 and CD31. Alexa Fluor 488- or 594-conjugated secondary antibodies were applied for 1 h at RT in the dark, and nuclei were counterstained with DAPI. Images were captured with an Axio Vert.A1 microscope, and the proportion of CCR5⁺ or CD31⁺ areas was quantified using ImageJ software and expressed as a percentage of total area analyzed. Quantification was based on five randomly selected fields per tissue section. Details of all antibodies used are provided in Table S2.

### Endothelial tube formation assay

The angiogenic capacity of NK-CM was evaluated using a Matrigel-based tube formation assay. Growth factor-reduced Matrigel (Corning, Cat# 354230; NY, USA) was thawed on ice, and 50 μL was dispensed into each well of a pre-chilled 96-well plate. After polymerization at 37 °C for 30 min, HUVECs were seeded at 2 × 10⁴ cells per well in 200 μL of treatment medium (control medium, pNK-CM, or drNK-CM). Recombinant human VEGF-165 (rVEGF; 10 ng/mL, PeproTech, Cat# 100-20) was used as a reference pro-angiogenic control. Cells were incubated for 6 h at 37 °C in 5% CO₂, and tube-like structures were imaged using an Axio Vert.A1 inverted microscope. Quantitative analysis of total tube length and branch point number was performed using ImageJ software.

### *In vivo* wound healing assay

Eight- to ten-week-old male C57BL/6J mice were randomly assigned to treatment groups (n = 6 *per* group) using a random number generator to ensure unbiased allocation. C57BL/6J mice were chosen as they are the most widely used strain for cutaneous wound healing studies, with well-documented immune and repair responses ensuring reproducibility and translational relevance. Treatment groups included: unconditioned medium control, PBS control, pNK-CM, drNK-CM, drNK-CCM, pNK-CM or drNK-CM combined with CCR1 (J113863, 10 μM), CCR3 (SB328437, 10 μM), or CCR5 (MVC, 10 μM) antagonists, or neutralizing antibodies against CCL3/4, CCL5, or the combination of CCL3/4/5. Unless otherwise specified, all CM treatments were applied at 5%, with drNK-CCM indicating concentrated drNK-CM. An additional positive control group received recombinant VEGF (25 μg/wound in PBS), freshly prepared and applied once daily for the first three days post-injury.

Mice were anesthetized by isoflurane inhalation (2-3% induction, 1.5-2% maintenance in oxygen) and dorsal hair was removed using a commercial depilatory cream (Reckitt Benckiser, Slough, UK). Two standardized, full-thickness excisional wounds (5 mm diameter) were generated on the dorsal skin using a sterile biopsy punch (Integra Miltex, Princeton, NJ, USA) to ensure uniform wound size, depth, and location. Immediately after wounding, 50 μL of conditioned medium, vehicle control, or inhibitor/antibody-containing solution were applied topically, with treatments repeated every other day for a total of three applications. Wounds were photographed on days 0, 3, 5, 7, and 10 using a fixed imaging setup (constant distance, lighting, and scale marker). All images were coded, and wound closure was quantified in ImageJ software under blinded conditions by independent investigators. Wound closure (%) was calculated as: Wound closure (%) = (area at day 0 - area at day X) / area 0 × 100.

### Histological evaluation of wound tissues

On day 10 post-injury, mice were euthanized, and wound tissues were harvested, rinsed in PBS, and fixed in 10% neutral buffered formalin (NBF; Sigma-Aldrich) at 4 °C overnight. Fixed tissues were processed using standard histological protocols, embedded in paraffin, and sectioned at 10 μm thickness with a rotary microtome (Leica RM2235; Leica Biosystems, Wetzlar, Germany). Sections were stained with hematoxylin and eosin (H&E; Abcam, ab245880) to assess tissue architecture and with Masson's trichrome Masson's trichrome (BioGnost, MST-100T, BioGnost Ltd., Medjugorskae, Zagreb, Croatia) to evaluate collagen deposition. Images were captured using an Axio Vert.A1 microscope, and quantitative analysis of re-epithelialization, epidermal thickness, and collagen content was performed using ImageJ software by blinded investigators. At least five randomly selected fields per section were analyzed for each sample.

### Statistical analysis

All quantitative data are presented as mean ± standard error of the mean (SEM), unless otherwise stated. For comparison between two groups, an unpaired, two-tailed Student's t-test was applied. For multiple group comparisons, one-way ANOVA with Tukey's post hoc test was used. Statistical significance was defined as follows: *p < 0.05 and **p < 0.01. All experiments were performed with at least three independent biological replicates. Statistical analyses were conducted using Microsoft Excel (Microsoft Corporation, Redmond, WA, USA).

## Results

### Direct reprogramming generates CD56^bright^CD16^bright^ NK cells with a distinct pro-regenerative secretome

**Figure 1:**
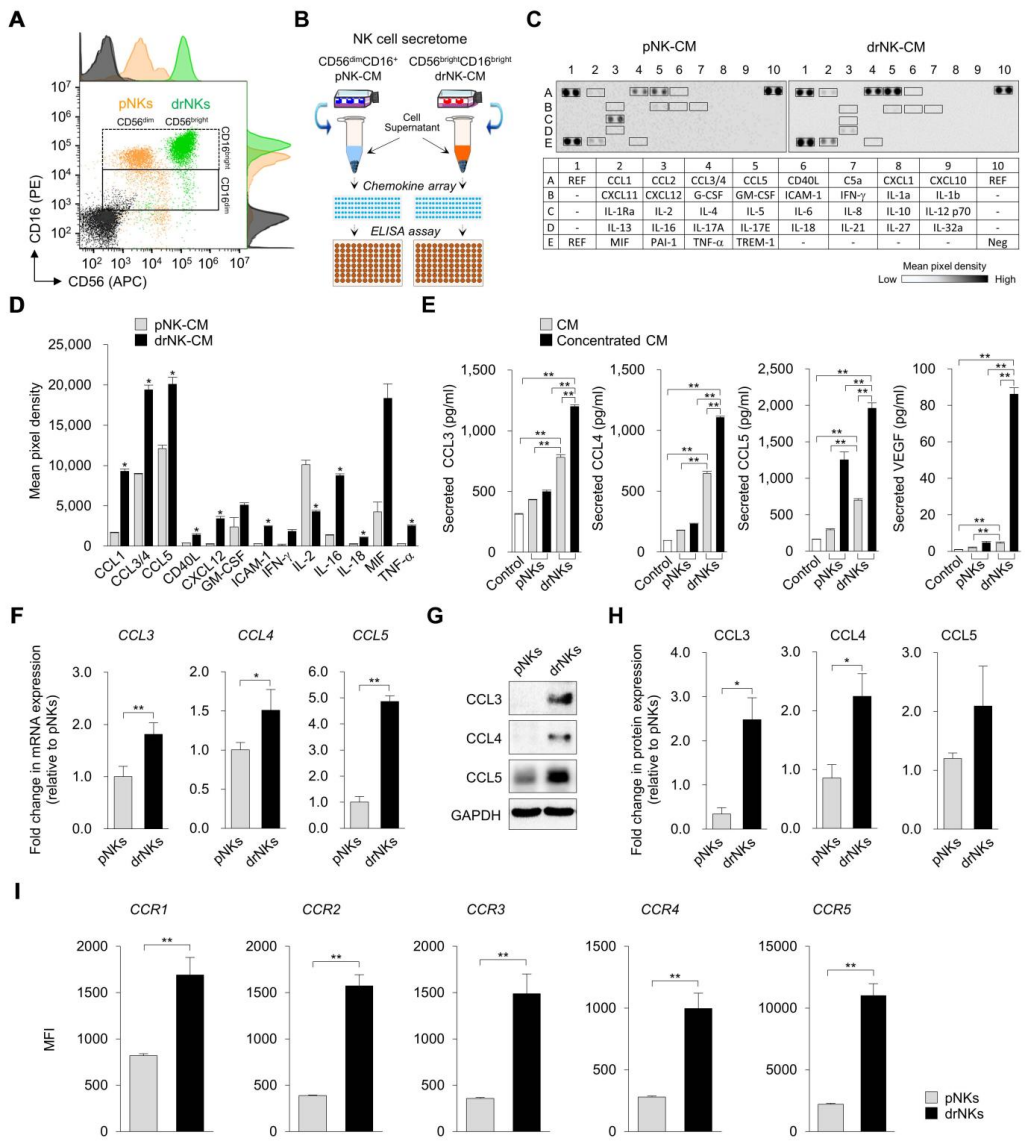
** Immunophenotypic and secretomic characterization of primary and directly reprogrammed NK cells. (A)** Flow cytometric analysis of CD56 and CD16 surface expression in PBMC-derived NK cells (pNKs) and directly reprogrammed NK cells (drNKs). **(B)** Schematic of the experimental workflow for chemokine profiling in NK cell-conditioned media (NK-CM), incorporating cytokine array and ELISA analyses. **(C)** Representative cytokine array membrane images showing comparative chemokine secretion profiles between pNK-CM and drNK-CM. Spot intensities reflect with chemokine abundance. **(D)** Densitometric quantification of selected chemokine signals from (C) normalized to internal reference (REF) controls. Data represent mean ± SEM (n = 3). **(E)** ELISA quantification of secreted CCL3, CCL4, CCL5, and VEGF levels in pNK-CM and drNK-CM. Data represent mean ± SEM (n = 5). **(F)** Relative mRNA expression of *CCL3*, *CCL4*, and *CCL5* in NK cell subsets, determined by qRT-PCR and normalized to *GAPDH*. Data represent mean ± SEM (n = 3). **(G)** Western blot analysis of chemokine protein expression in pNKs and drNKs. **(H)** Densitometric quantification of protein levels from (G). Data represent mean ± SEM (n = 3). **(I)** Flow cytometric evaluation of chemokine receptor expression (CCR1-CCR5) in pNKs and drNKs (mean fluorescence intensity, MFI). Data represent mean ± SEM (n = 3). Statistical comparisons were performed using two-tailed Student's t-tests. *p < 0.05, **p < 0.01 compared with indicated control groups. Horizontal brackets denote pairwise comparisons.

Flow cytometry analysis revealed clear phenotypic differences between PBMC-derived NK cells (pNKs) and directly reprogrammed NK cells (drNKs) (Figure 1A). While pNKs mainly exhibited the CD56^dim^CD16^+^ phenotype characteristic of cytotoxic NK subsets, OSKM (OCT4, SOX2, KLF4, MYC)-mediated reprogramming 30 produced drNKs with a stable CD56^bright^CD16^bright^ profile, a phenotype linked to enhanced secretory activity and immunomodulatory capacity 31, 32. This phenotype was maintained through day 42 of culture under feeder-free conditions (Figure S1). As anticipated, drNKs displayed negligible cytotoxicity against normal human epidermal keratinocytes (HEKs), dermal fibroblasts (HDFs) and umbilical vein endothelial cells (HUVECs), while retaining robust activity toward malignant K562 cells, as measured by CD107a degranulation (Figure S2A-B) and granzyme B release (Figure S2C). These results demonstrate that drNKs preserve selective antitumor activity without inducing nonspecific cytotoxicity toward normal stromal or epithelial cells.

Global transcriptomic profiling revealed that drNKs acquired a distinct gene expression program compared with pNKs (Figure S3A). Of 2,520 secretome-associated genes annotated in the Human Protein Atlas, 450 were significantly differentially expressed between drNKs and pNKs (FDR < 0.05), underscoring a broad reprogramming of the secretory landscape (Figure S3A). Genes upregulated in drNKs included pro-inflammatory and immunoregulatory cytokines such as *TNF*, *IL18*, *IFNG*, and *CD40LG*, along with secretory regulators (*FAM3C*, *TXLNA*) and stromal-activation mediators (*GREM2*, *CSF1*) (Figure S3B). In contrast, canonical cytotoxic mediators (*GZMB*, *PRF1*) were downregulated, indicating a shift away from dominant cytotoxicity toward a program integrating immune modulation and tissue repair.

Chemokine profiling highlighted robust expression of *CCL3*, *CCL4*, *CCL5*, the principal ligands of CCR5 that regulate fibroblast activation, immune chemotaxis, and endothelial migration (Figure S3C). Notably, drNKs also upregulated chemokines *XCL1* (XC chemokine family), *CCL18* (CC family), *PF4*/*CXCL4*, and *PF4V1*/*CXCL4L1* (CXC family), which are implicated in mucosal defense, monocyte and T-cell recruitment, platelet-driven vascular remodeling, and vascular stabilization. At the receptor level, drNKs expressed higher levels of *IL2RG*, *IL2RB*, *IL1RL1*, *IL18R1*, and *IL12RB2* (Figure S3D, enhancing their responsiveness to γc cytokines, IL-18, and IL-12, all key drivers of NK-immune crosstalk. Furthermore, chemokine receptors *CCR1*, *CCR2*, *CCR5, CCR6*, and *CXCR3* were upregulated (Figure S3E), indicating that drNKs are primed to both sense and respond to stromal and inflammatory cues.

Gene ontology (GO) enrichment analysis corroborated these findings, revealing biological processes enriched for immune response, cytokine-mediated signaling, ECM organization, wound healing, and angiogenesis (Figure S3F). Cellular component enrichment emphasized extracellular region, collagen-containing ECM, exosomes, and endoplasmic reticulum lumen (Figure S3G), consistent with a highly secretory phenotype. Molecular function categories included cytokine activity, receptor binding, and ECM structural components (Figure S3H). KEGG pathway enrichment further demonstrated activation of cytokine-cytokine receptor interactions, chemokine signaling, PI3K-Akt signaling, ECM-receptor interactions, lysosome pathways, and hematopoietic lineage programs (Figure S3I). These transcriptomic changes were validated by qRT-PCR (Figure 1F), immunoblotting (Figure 1G-H), and flow cytometry (Figure 1I). Together, these analyses confirm that drNKs acquire an integrated immune-regenerative transcriptomic program.

Consistent with the transcriptomic data, cytokine array analysis demonstrated marked enrichment of pro-regenerative factors in drNK-conditioned medium (drNK-CM) compared with pNK-CM (Figure 1B-D). Prominent increases included CCL1 (~5.6-fold), CCL3/4 (~2.2-fold), CCL5 (~1.7-fold), CD40L (~3.5-fold), CXCL12 (~13.6-fold), GM-CSF (~2.1-fold), ICAM-1 (~8.2-fold), IFN-γ (~18.5-fold), IL-16 (~2.8-fold), MIF (~4.3-fold), and TNF-α (~8.3-fold), while IL-2 was decreased (~0.43-fold). This secretome profile indicates a shift from canonical cytotoxic activity toward immunoregulatory and reparative signaling. ELISA further confirmed that dose-dependent increases in CCL3, CCL4, CCL5, and VEGF in both drNK-CM and concentrated drNK-CM (drNK-CCM) (Figure 1E). Specifically, CCL3 rose ~1.8-2.2-fold, CCL4 ~2.4-2.6-fold, and CCL5 ~2.4-2.8-fold compared with pNK-CM, while VEGF increased ~2.2-fold in drNK-CM and ~18-fold in drNK-CCM. Western blotting corroborated elevated CCL3/4/5 protein levels in drNKs relative to pNKs (Figure 1G-H). In parallel, flow cytometry showed increased surface expression of CCR1-5 (Figure 1I), underscoring the potential for both paracrine and autocrine CCR5-driven signaling. Collectively, these findings establish that OSKM-driven reprogramming yields a phenotypically and functionally distinct NK subset characterized by enrichment of the CCL3/4/5-CCR5 axis and a secretome primed for regenerative activity.

### drNK-CM promotes keratinocyte and fibroblast proliferation and migration via the CCL5-CCR5 signaling axis

**Figure 2:**
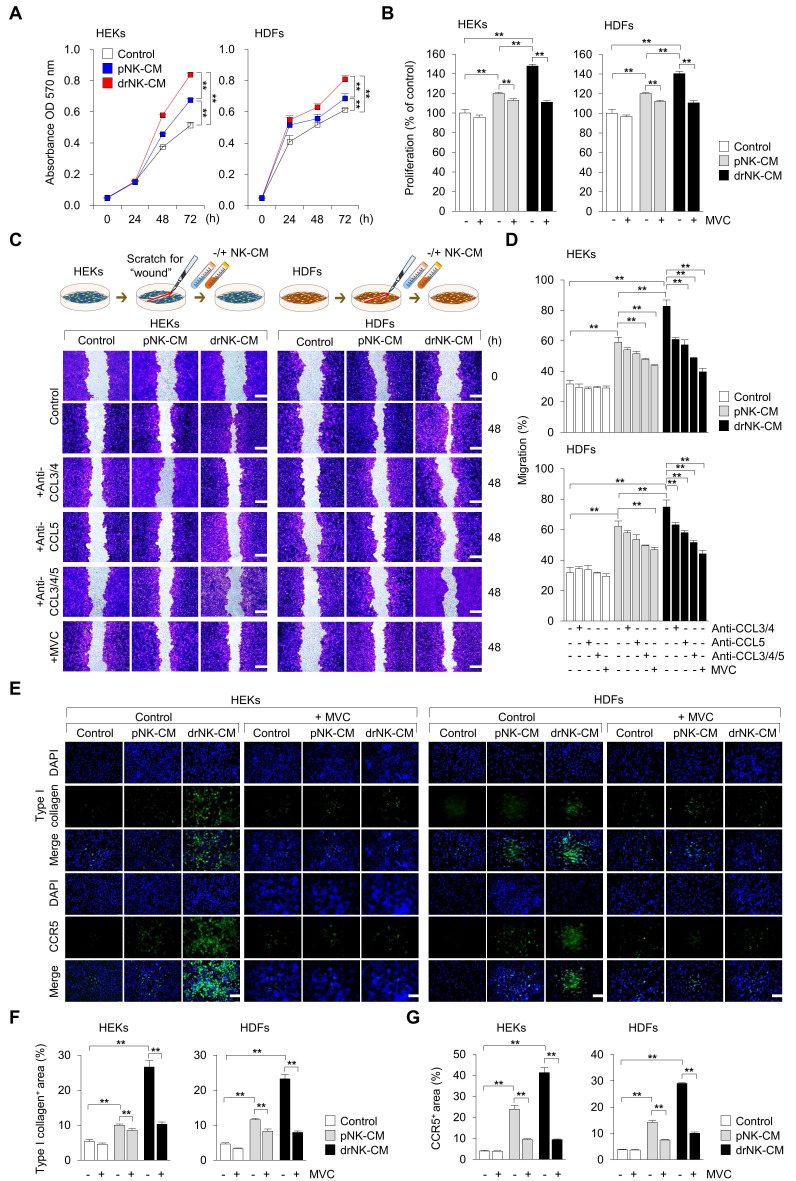
** drNK-CM enhances proliferation, migration, and matrix remodeling in keratinocytes and dermal fibroblasts. (A)** Time-course MTT assay of cell proliferation in human epidermal keratinocytes (HEKs) and dermal fibroblasts (HDFs) treated with control medium, 5% pNK-CM, or 5% drNK-CM. Data represent mean ± SEM (n = 3). **(B)** Quantification of cell proliferation at 48 h with or without CCR5 antagonist maraviroc (MVC; 10 µM). Data represent mean ± SEM (n = 5). **(C)** Representative crystal violet-stained images from scratch assays of HEKs and HDFs treated with 5% pNK-CM or 5% drNK-CM in the presence or absence of anti-CCL3/4, anti-CCL5, or anti-CCL3/4/5 antibodies (10 ng/mL), or MVC (10 µM). Images at 0 and 48 h are shown. Scale bar = 50 μm. **(D)** Quantification of HEK and HDF migration (%) at 48 h based on scratch gap closure from (C). Data represent mean ± SEM (n = 5). **(E)** Immunofluorescence staining of Type I collagen and CCR5 expression in HEKs and HDFs following treatment. Scale bars = 100 μm. **(F-G)** Quantification of COL1A1^+^ and CCR5^+^ fluorescence intensity signals normalized to DAPI. Data represent mean ± SEM (n = 3). Statistical analysis was performed by one-way ANOVA with Tukey's post hoc test; *p < 0.05, **p < 0.01 compared with indicated control groups.

Given the robust enrichment of CCL3, CCL4, and CCL5 in drNK-CM, we next investigated its functional effects on skin-resident cells central to wound repair. MTT assays revealed that drNK-CM significantly enhanced proliferation of both human epidermal keratinocytes (HEKs) and dermal fibroblasts (HDFs) compared with pNK-CM (Figure 2A-B). Dose-response analysis identified 5% CM as the optimal concentration, with diminished efficacy observed at 10-20%, likely due to cytokine-induced feedback inhibition or cytotoxic stress (Figure S4). At this concentration, drNK-CM increased proliferation to 147.9 ± 1.8% in HEKs and 140.4 ± 2.3% in HDFs, whereas pNK-CM induced only120.1 ± 0.9% and 120.2 ± 0.9%, respectively (Figure 2B). To determine whether this effect was mediated by CCR5, the primary receptor for CCL3/4/5, both cell types were co-treated with the CCR5 antagonist maraviroc (MVC). MVC significantly abolished the proliferation response, confirming that CCR5 signaling play a central role in drNK-CM-induced proliferation.

Scratch wound healing assays further showed that drNK-CM markedly accelerated migration in both HEKs (82.7 ± 4.1%) and HDFs (74.9 ± 4.6%) compared with pNK-CM (59.1 ±3.3% and 62.2 ± 3.5%) and control medium (31.7± 2.3% and 31.6 ± 3.5%) (Figure 2C-D). Neutralization experiments dissected ligand contributions: blockade of CCL3/4 reduced closure to 61.0 ± 1.2% in HEKs and 63.1 ± 1.5% in HDFs, CCL5 blockade further decreased migration to 57.3 ± 3.3% and 58.1 ± 1.5%, and combined CCL3/4/5 blockade suppressed migration to 48.7 ± 0.6% and 51.6 ± 1.3%. MVC treatment produced the strongest inhibition (39.7 ± 2.2% in HEKs; 44.2 ± 2.3% in HDFs), underscoring the broader effect of receptor-level blockade compared with ligand-specific inhibition.

Immunofluorescence analysis confirmed that drNK-CM increased both CCR5 expression and extracellular matrix deposition. In HEKs, the proportion of COL1A1⁺ cells increased from 5.4 ± 0.5% in controls and 10.0 ± 0.3% with pNK-CM to 26.6 ± 1.8% with drNK-CM, while CCR5⁺ area rose from 4.0 ± 0.3% and 23.8 ± 1.8% to 41.3 ± 2.3%, respectively. HDFs showed a similar pattern: COL1A1⁺ cells rose from 7.2 ± 0.6% (control) and 12.4 ± 0.5% (pNK-CM) to 29.8 ± 2.0% (drNK-CM), and CCR5⁺ area expanded from 5.6 ± 0.4% and 21.7 ± 1.5% to 39.1 ± 2.1% (Figure 2E-G). Importantly, these effects were reversed by MVC, whereas CCR1 (J113863) or CCR3 (SB328437) antagonists had no significant impact (Figure S5). Cell viability assays confirmed that none of the treatments exerted cytotoxic effects (Figure S6). Together, these results demonstrate that drNK-CM promotes keratinocyte and fibroblast proliferation, migration, and ECM remodeling through CCR5 signaling, with CCL3, CCL4, and CCL5 providing cooperative inputs. The more pronounced inhibition by MVC compared with individual ligand blockade highlights the broader regulatory effect of receptor-level antagonism.

### drNK-CM induces extracellular matrix remodeling via coordinated collagen synthesis and MMP activation

Having demonstrated the proliferative and migratory effects of drNK-CM on skin-resident cells, we next evaluated whether these functional outcomes were accompanied by ECM remodeling, an essential process for re-epithelialization, angiogenesis, and structural reorganization during wound healing 33, 34. We assessed matrix-associated genes and proteins in HEKs and HDFs following treatment with drNK-CM, pNK-CM, or control medium.

**Figure 3:**
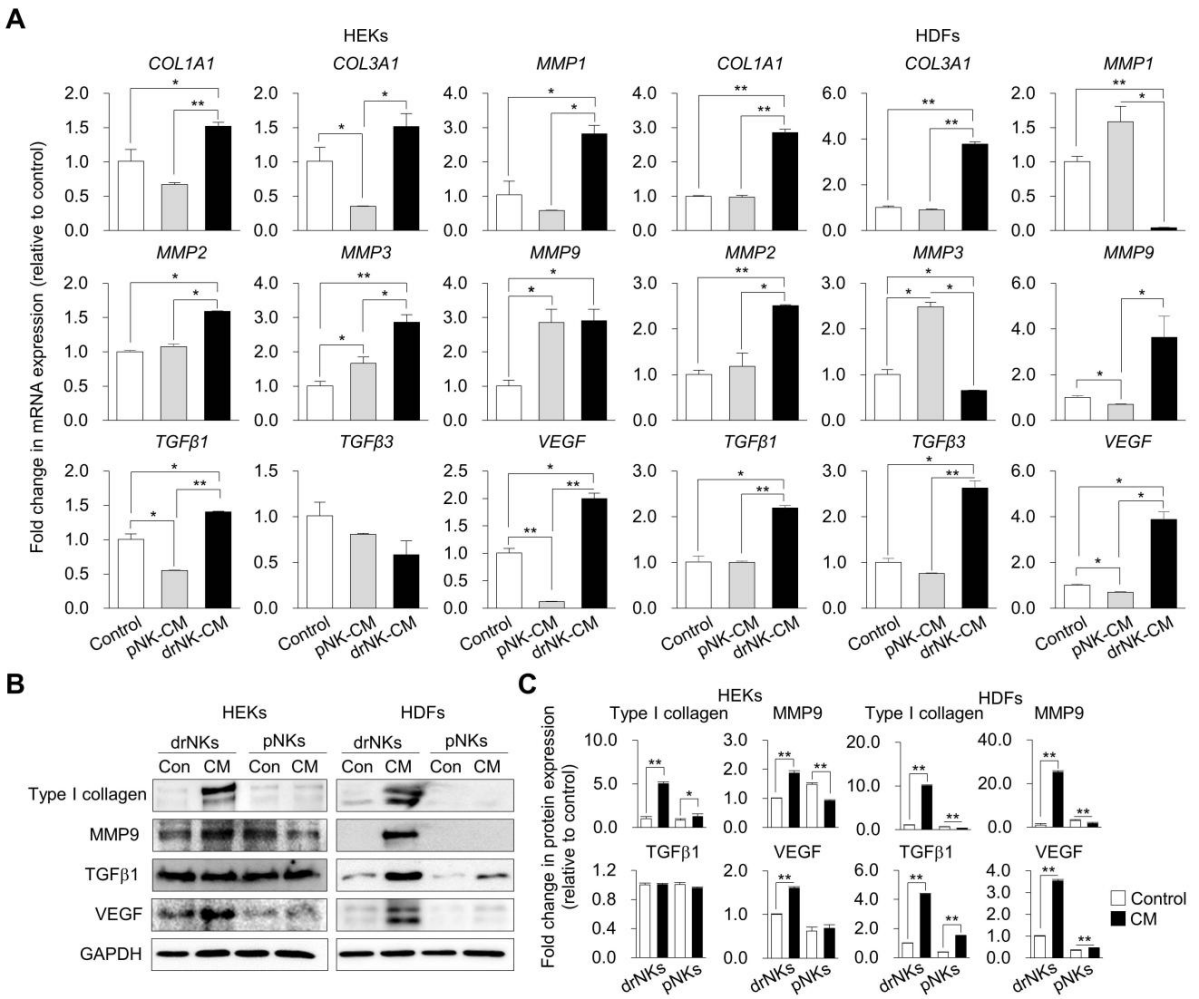
** drNK-CM modulates gene and protein expression associated with wound healing in skin-resident cells. (A)** qRT-PCR analysis of ECM-associated genes (*COL1A1*, *COL3A1)*, matrix metalloproteinases (*MMP1*, *MMP2*, *MMP3*, *MMP9*), and pro-regenerative growth factors (*TGFβ1*, *TGFβ3*, *VEGF*) in HEKs and HDFs treated with control, 5% pNK-CM, or 5% drNK-CM. Gene expression levels were normalized to *GAPDH*. **(B)** Western blot analysis of Type I collagen, MMP9, TGFβ1, and VEGF proteins in cells treated under the same conditions. GAPDH served as loading control. **(C)** Densitometric quantification of protein levels from (B). Data represent mean ± SEM (n = 3). Statistical analysis was performed by one-way ANOVA with Tukey's post hoc test; *p < 0.05, **p < 0.01 compared with indicated control groups.

Quantitative RT-PCR revealed that drNK-CM markedly upregulated ECM-associated transcripts in both cell types (Figure 3A). In HEKs, expression of *COL1A1* and *COL3A1* (type I and III collagens), *MMP1*, *MMP2*, *MMP3*, *MMP9*, *TGFβ1*, and *VEGF* was significantly elevated, whereas *TGFβ3* was consistently downregulated, suggesting a remodeling profile favouring repair over fibrosis. In HDFs, drNK-CM similarly induced strong upregulation of *COL1A1*, *COL3A1*, *MMP2*, *MMP9*, *TGFβ1*, *TGFβ3*, and *VEGF*. By contrast, pNK-CM more prominently increased *MMP1* and *MMP3*, indicating differences in the balance of matrix synthesis and degradation between the two secretomes.

Western blot analysis confirmed these transcriptional trends (Figure 3B-C). In HEKs, drNK-CM significantly elevated COL1A1, MMP9, and VEGF protein levels, whereas in HDFs, COL1A1, MMP9, TGFβ1, and VEGF were upregulated. These results show that drNK-CM not only promotes collagen synthesis but also activates MMP-dependent matrix remodeling and angiogenic signaling. Taken together, these findings indicate that drNK-CM orchestrates a balanced ECM program, coupling collagen synthesis with proteolytic turnover, thereby generating a reparative microenvironment that complements its mitogenic and motogenic activities and promotes efficient tissue repair and revascularization.

### drNK-CM promotes endothelial proliferation, migration, and angiogenesis through cooperative activation of the CCR5 signaling axis

**Figure 4:**
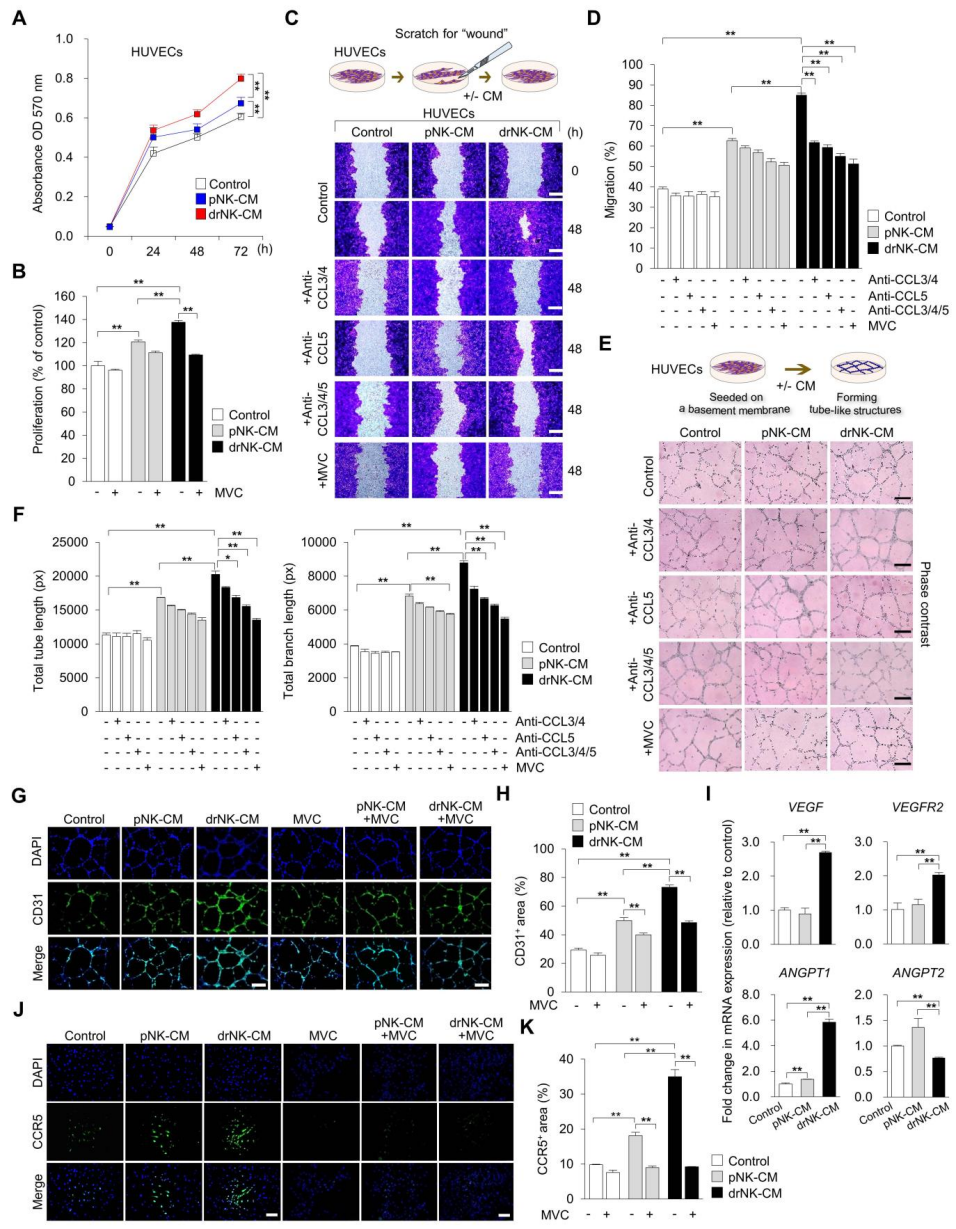
** drNK-CM enhances endothelial proliferation, migration, and angiogenic activity. (A)** Time-course MTT assay of proliferation in HUVECs treated with control medium, 5% pNK-CM, or 5% drNK-CM. Data represent mean ± SEM (n = 3). **(B)** Quantification of HUVEC proliferation at 48 h with or without MVC (10 μM). Data represent mean ± SEM (n = 3). **(C)** Representative scratch assay images of HUVECs treated with 5% pNK-CM or drNK-CM in the presence or absence of anti-CCL3/4, anti-CCL5, or anti-CCL3/4/5 antibodies (10 ng/mL), or MVC (10 µM). Scale bar = 50 μm. **(D)** Quantification of HUVEC migration (%) at 48 h based on scratch gap recovery from (C). Data represent mean ± SEM (n =5). **(E)** Representative tube formation assay images in HUVECs treated with control medium, pNK-CM, or drNK-CM, with or without anti-CCL3/4, anti-CCL5, or anti-3/4/5 antibodies (10 ng/mL), or MVC (10 µM). Scale bar = 100 μm. **(F)** Quantification of total tube length (left) and branch points (right) from (E). Data represent mean ± SEM (n = 3). **(G)** Immunofluorescence staining for CD31 in tube-forming HUVECs. Scale bar = 100 μm. **(H)** Quantification of CD31⁺ network area. **(I)** qRT-PCR analysis of angiogenesis-related genes (*VEGF*, *VEGFR2*, *ANGPT1*, *ANGPT2*) in HUVECs under the indicated conditions. Data represent mean ± SEM (n = 3). **(J)** Immunofluorescence staining of CCR5 in HUVECs treated with or without MVC. Scale bar = 100 μm. **(K)** Quantification of CCR5 fluorescence intensity from (J). Data are presented as mean ± SEM (n = 3). One-way ANOVA with Tukey's post-hoc test was used; *p < 0.05, **p < 0.01 compared with indicated control groups.

Given the marked enrichment of CCR5-binding chemokines (CCL3, CCL4, CCL5) in the drNK secretome, we next examined the ability of drNK-CM to modulate endothelial cell behaviors relevant to neovascularization. HUVECs treated with 5% drNK-CM displayed significantly increased proliferation compared to pNK-CM or control groups (Control: 100 ± 3.9%; pNK-CM: 120.8 ± 1.4%; drNK-CM: 137.7 ± 1.6%) (Figure 4A-B), consistent with mitogenic effects previously observed in keratinocytes and fibroblasts. A dose-response analysis confirmed 5% CM as the optimal concentration for stimulating HUVEC proliferation (Figure S4).

Scratch wound assays demonstrated that drNK-CM markedly enhanced HUVEC migration (85 ± 4.0% closure) compared with both pNK-CM (62.7 ± 2.2%) and control medium (39.0 ± 1.4%) (Figure 4C-D). Neutralization of CCL3/4 reduced migration to 61.8 ± 0.69%, while CCL5 blockade further decreased migration to 59.4 ± 1.33%. Combined CCL3/4/5 blockade lowered migration to 55.0 ± 1.4%, and MVC treatment produced the strongest suppression at 51.3 ± 2.2%. Consistent with migration data, Matrigel-based tube formation assays show that drNK-CM markedly enhance angiogenic morphogenesis. HUVECs exposed to drNK-CM displayed significantly greater total tube length (20,268 ± 476 px) and branch length (8,790 ± 112 px) compared with pNK-CM (16,839 ± 37 px, 6,812 ± 126 px) and control medium (11,320 ± 268 px, 3,875± 33 px) (Figure 4E-F). Neutralization of CCL3/4 partially reduced tube length (18,335 ± 128 px) and branch length (7,237 ± 175 px), whereas CCL5 blockade produced a stronger decrease (16,854 ± 308 px, 6,668 ± 72 px). Combined blockade of CCL3/4/5 caused further suppression (15,523 ± 238 px, 6,270 ± 70 px), while MVC treatment led to the greatest reduction (13,545 ± 226 px, 5,477 ± 87 px). Together, these results show that drNK-CM-induced motility and angiogenesis are regulated by CCR5 signaling through cooperative actions of CCL3, CCL4, and CCL5. MVC exerts broader suppression by blocking the receptor and potentially disrupting downstream CCR5-associated signaling pathways.

In direct comparison with recombinant VEGF (rVEGF, 10 ng/mL), a widely studied pro-angiogenic factor, drNK-CM (5%) outperformed VEGF in promoting angiogenesis *in vitro*, as evidenced by significantly greater tube length and branch formation in HUVEC assays (Figure S7A-B). Immunofluorescent staining for CD31 confirmed increased microvascular network formation, with CD31⁺ area rising from 29.3 ± 1.3% (control) and 49.9 ± 1.9% (pNK-CM) to 73.13 ± 1.5% (drNK-CM) (Figure 4G-H). This effect was abrogated by MVC, reinforcing the essential role of CCR5-mediated signaling.

At the molecular level, qRT-PCR revealed upregulation of key angiogenic genes in drNK-CM-treated HUVECs, including *VEGF* (2.69 ± 0.03-fold), *VEGFR2* (2.02 ± 0.04-fold), and *ANGPT1* (5.82 ± 0.17-fold), along with modest downregulation of *ANGPT2* (0.76 ± 0.01-fold) (Figure 4I). This expression profile supports the induction of a stabilized, pro-angiogenic endothelial phenotype. Notably, CCR5 expression itself was elevated in response to drNK-CM, as demonstrated by immunofluorescence (Figure 4J-K), and this induction was suppressed by MVC, confirming ligand-dependent receptor regulation. Collectively, these findings demonstrate that drNK-CM promotes endothelial proliferation, migration, and capillary-like network formation through CCR5-dependent mechanisms. The partial inhibition of function by CCL5 neutralization, in contrast to the more complete suppression by MVC, highlights a cooperative model wherein CCL3, CCL4, and CCL5 synergistically engage CCR5 to orchestrate angiogenic activation. These coordinated responses position drNK-CM as a potent secretome for vascular remodeling and regeneration in tissue repair contexts.

### drNK-CM accelerates cutaneous wound healing *in vivo* via CCR5-dependent regenerative programming

**Figure 5:**
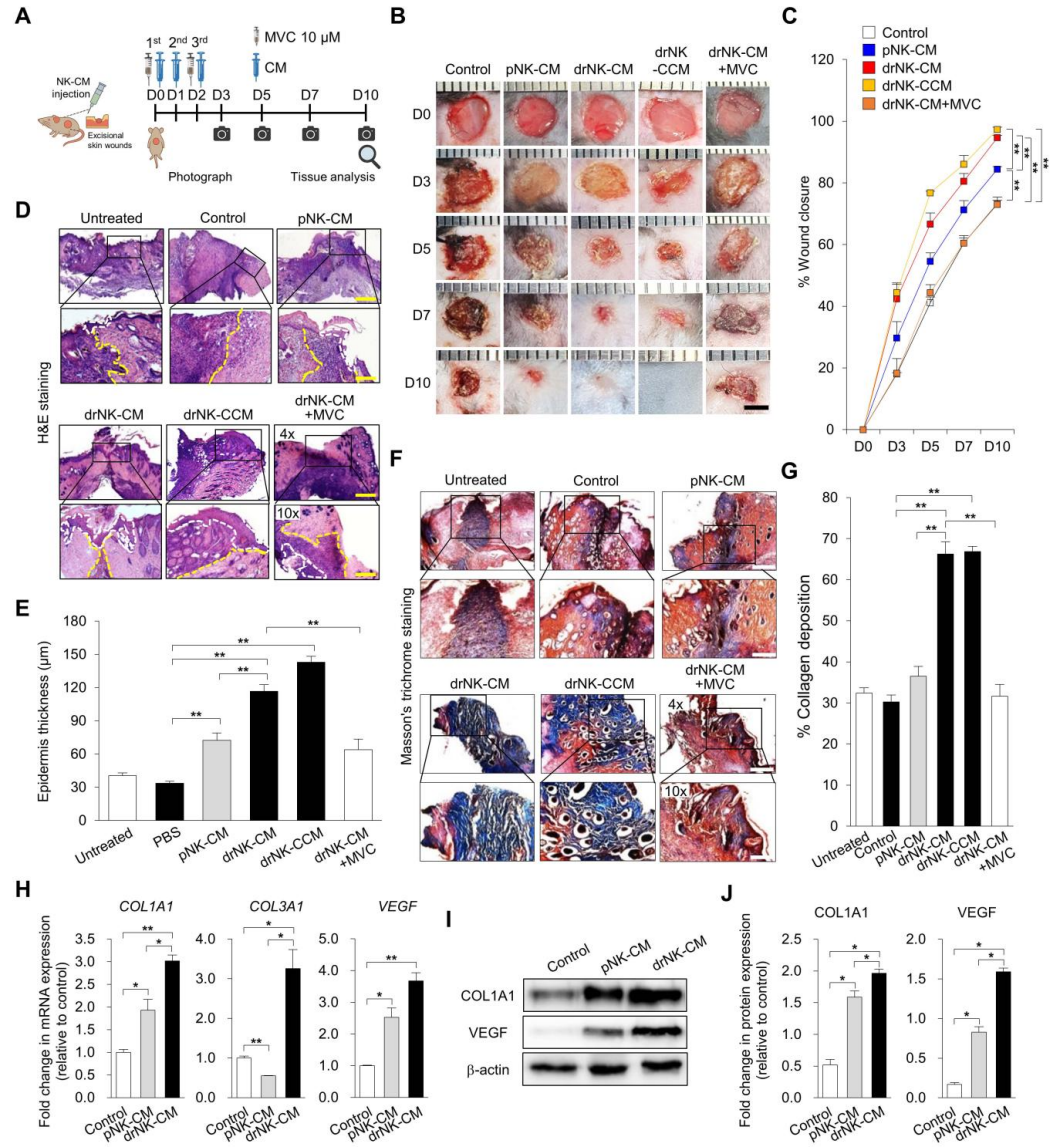
** drNK-CM accelerates *in vivo* wound healing and promotes skin regeneration. (A)** Schematic of full-thickness excisional wound model in C57BL/6J mice. Wounds were treated by topical application of control medium, pNK-CM, drNK-CM, concentrated drNK-CM (drNK-CCM), or drNK-CM with MVC (10 µM). **(B)** Representative wound images and **(C)** Quantitation of wound closure (%) over time. Data represent mean ± SEM (n = 3). **(D)** Hematoxylin and Eosin staining of wound sections at day 7 showing re-epithelialization (dashed lines). Scale bar = 100 μm. **(E)** Quantification of epidermal thickness from (D). Data represent mean ± SEM (n = 3). **(F)** Masson's trichrome staining of collagen deposition at day 7. Scale bar = 100 μm. **(G)** Quantification of collagen^+^ area from (F). Data represent mean ± SEM (n = 5). **(H)** qRT-PCR analysis of *COL1A1*, *COL3A1* and *VEGF* expression in wound tissue. Data represent mean ± SEM (n = 3). **(I)** Western blot analysis of COL1A1 and VEGF protein levels. **(J)** Densitometric quantification of protein levels from (I). Data represent mean ± SEM (n = 4 mice per group). One-way ANOVA with Tukey's post hoc test; *p < 0.05, **p < 0.01 compared with indicated controls. Mice were randomized; wound closure assessment was performed blinded.

To assess the therapeutic efficacy of drNK-CM *in vivo*, we used a full-thickness excisional wound model in C57BL/6J mice, in which two 5-mm circular wounds were created on the dorsal skin and treated topically once daily with control medium, pNK-CM, drNK-CM, concentrated drNK-CM (drNK-CCM), or drNK-CM in combination with antagonists of CCR1 (J113863), CCR3 (SB328437), or CCR5 (MVC), or with neutralizing antibodies against CCL3/4 and CCL5 individually or in combination (Figure 5A). Wounds treated with drNK-CM closed significantly faster than those treated with control medium or pNK-CM (Figure 5B-C). By day 5, closure reached 66.6 ± 3.6% with drNK-CM and 76.8 ± 0.5% with drNK-CCM, compared with 41.1 ± 1.9% in control and 54.6 ± 2.7% in pNK-CM, and by day 10, closure was nearly complete with both drNK-CM (94.7 ± 3.6%) and drNK-CCM (97.3 ± 0.7%), whereas control and pNK-CM groups reached only 73.3 ± 1.0% and 84.5 ± 0.8%, respectively (Figure 5B-C).

To dissect the underlying mechanism, we performed receptor and ligand blockade assays. CCR1 (J113863) or CCR3 (SB328437) inhibition produced minimal changes in wound closure, whereas CCR5 blockade markedly impaired the regenerative benefit (Figure 5B-C, S8B-C). Neutralization antibody experiments further clarified the contribution of individual CCR5 ligands. At day 10, wounds treated with drNK-CM closed by 90.5 ± 2.2%, compared with 49.7 ± 2.0% in control. Blockade of CCL3/4 reduced closure to 64.6 ± 2.2%, while CCL5 inhibition further decreased it to 59.6 ± 0.6% (Figure S9). Combined neutralization of CCL3/4/5 led to 56.1 ± 2.0% closure, closely mirroring the suppression seen with CCR5 antagonism. These results indicate that while CCL5 is a dominant ligand, the cooperative input of CCL3 and CCL4 enhances regeneration, and that receptor-level inhibition exerts the broadest suppression by blocking multi-ligand signaling and downstream CCR5 pathways.

Histological analysis supported these functional findings. Hematoxylin and eosin (H&E) staining revealed that drNK-CM accelerated re-epithelialization and dermal restoration compared with pNK-CM or control, while MVC treatment delayed epithelial coverage and produced disorganized granulation tissue (Figure 5D-E). Importantly, drNK-CCM induced even greater epithelial thickening and dermal organization than standard drNK-CM, consistent with its superior wound closure activity. In contrast, inhibition of CCR1 with J113863 or CCR3 with SB328437 did not significantly alter epithelial repair, indicating that these pathways contribute little to the regenerative effects of drNK-CM (Figure 5D-E, S8D-E). Masson's trichrome staining further demonstrated enhanced collagen deposition in both drNK-CM and drNK-CCM groups, with drNK-CCM showing the most robust remodeling (Figure 5F-G). MVC treatment markedly reduced collagen accumulation, whereas J113863 and SB328437 again had negligible impact (Figure 5F-G, S8F-G).

At the molecular level, drNK-CM significantly upregulated the expression of *COL1A1*, *COL3A1*, and *VEGF* transcripts, markers for extracellular matrix remodeling and angiogenesis, compared with pNK-CM or control (Figure 5H). These gene-level changes were reflected in protein expression profiles, as Western blot analysis showed increased levels of COL1A1 and VEGF in the drNK-CM group (Figure 5I-J). When compared with rVEGF, drNK-CM demonstrate superior efficacy, accelerating wound closure (Figure S10A-B), enhancing epidermal thickness, and promoting more organized collagen remodeling (Figure S10C-D). Thus, whereas rVEGF supports angiogenesis, drNK-CM delivers broader regenerative benefits as a multifunctional secretome. Together, these findings demonstrate that drNK-CM promotes wound closure, re-epithelialization, collagen remodeling, and angiogenesis primarily through CCR5 signaling, driven by the cooperative actions of CCL3, CCL4, and CCL5.

### drNK-CM orchestrates CCR5-driven immune and endothelial activation to support tissue regeneration

**Figure 6:**
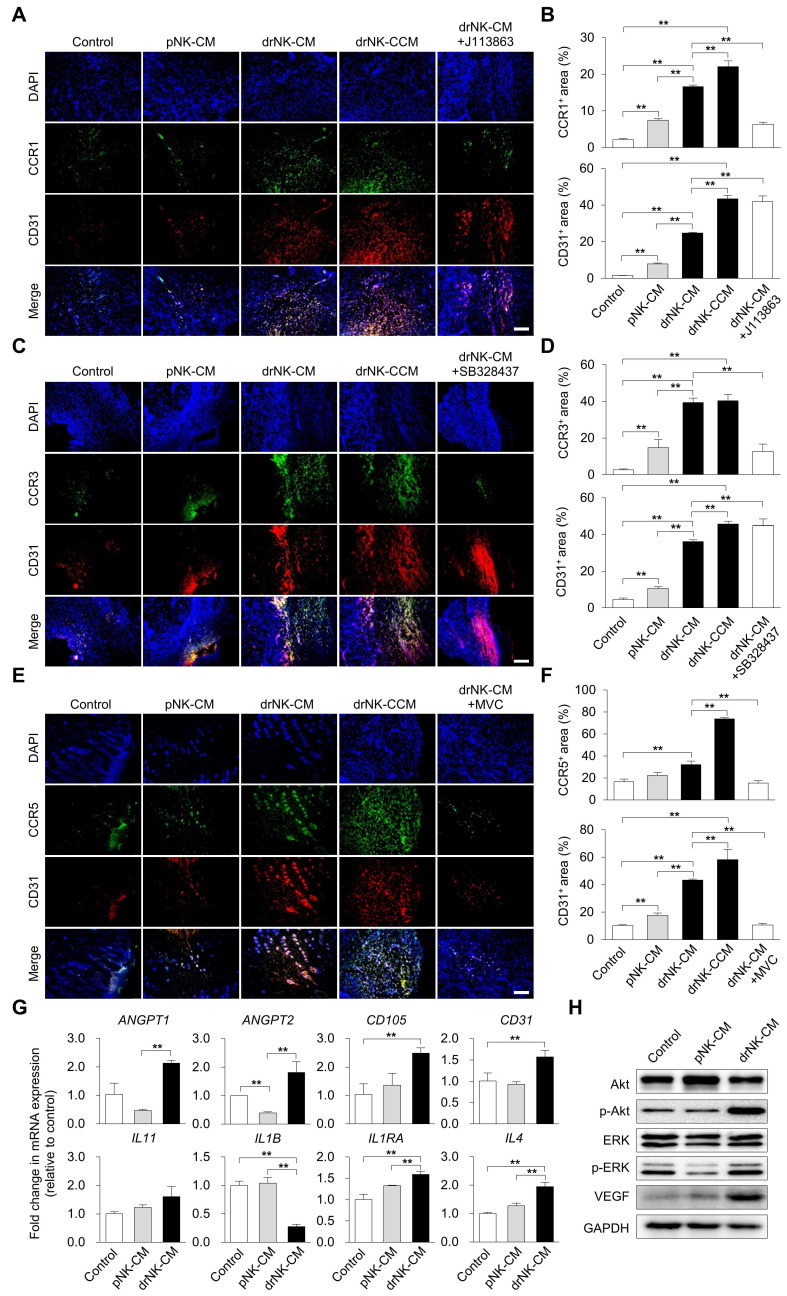
**drNK-CM upregulates CCR5 and CD31 expression and activates downstream regenerative signaling *in vivo*. (A, C, E)** Immunohistochemical staining of wound sections for CCR1/CD31 (A), CCR3/CD31 (C), and CCR5/CD31 (E) at day 10 post-injury. Wounds were treated by local injection of control medium, pNK-CM, drNK-CM, drNK-CCM, or drNK-CM with MVC (10 µM). Scale bar = 100 μm. **(B, D, F).** Quantitative analysis of CCR1^+^, CCR3^+^, and CCR5^+^/CD31^+^ regions. Data represent mean ± SEM (n = 4 mice per group). **(G)** qRT-PCR analysis of angiogenesis- and inflammation-related genes in wound tissues. Data are presented as mean ± SEM (n = 3 mice per group). **(H)** Western blot of total and phosphorylated AKT and ERK, and VEGF expression. Statistical analysis was performed by one-way ANOVA with Tukey's post hoc test; *p < 0.05, **p < 0.01 compared with indicated control groups. All tissue analyses were performed on randomized, blinded samples.

To elucidate the mechanistic underpinnings of drNK-CM-mediated regeneration, we assessed immune cell recruitment and endothelial activation in wound tissues. Immunohistochemical staining revealed significantly increased infiltration of CCR1⁺, CCR3⁺, and CCR5⁺ cells in wounds treated with drNK-CM and drNK-CCM compared to pNK-CM or control (Figure 6A-F). Pharmacological blockade of CCR1 and CCR3 with selective antagonists reduced CCR1⁺ and CCR3⁺ cell infiltration, respectively, without altering CD31⁺ endothelial cell density (Figure 6A-D). These findings suggest a role for CCR1 and CCR3 in immune cell recruitment but not in vascular remodeling.

In contrast, CCR5⁺ cell infiltration and CD31⁺ neovessel density were markedly enhanced in the drNK-treated groups and abrogated by co-treatment with MVC (Figure 6E-F). This indicates that CCR5 signaling is a central mediator of both immune and endothelial activation induced by drNK-CM. Concordantly, qRT-PCR analysis of wound tissue showed significant upregulation of angiogenesis-related genes (*ANGPT1*, *ANGPT2*, *CD105*, *CD31*) and immune-regulatory cytokines (*IL11*, *IL1B*, *IL1RN*, *IL4*) in response to drNK-CM (Figure 6G), implicating a dual role in promoting vascular growth and orchestrating a regenerative immune milieu.

Western blot analysis further supported these findings, revealing increased VEGF protein expression along with enhanced phosphorylation of AKT and ERK in drNK-CM-treated wounds (Figure 6H). These kinases are key effectors of endothelial cell proliferation, survival, and angiogenesis. Taken together, these data provide compelling evidence that drNK-CM drives cutaneous tissue regeneration by activating CCR5-dependent signaling pathways that coordinate immune infiltration, endothelial expansion, and pro-regenerative cytokine expression.

## Discussion

This study demonstrates that directly reprogrammed natural killer cells (drNKs), generated by OSKM-mediated conversion, acquires a distinct secretome enriched in chemokines and cytokines with pro-regenerative potential. Conditioned medium from these cells (drNK-CM) promotes epithelial and stromal proliferation, extracellular matrix (ECM) remodeling, angiogenesis, and immunomodulation through CCR5-centered paracrine signaling. These findings establish drNK-CM as a scalable and clinically translatable, cell-free therapeutic platform for tissue repair.

Traditionally, NK cells have been defined by their cytotoxicity and immune surveillance functions 15, 35, 36. Our data broaden this view by showing that drNKs exhibit reparative properties aligned with tissue-resident NK subsets (trNKs) 37-40. Unlike natural trNKs, which are rare and difficult to isolate, drNKs reproducibly acquire a CD56^bright^CD16^bright^ phenotype under feeder-free conditions and show negligible cytotoxicity toward healthy epithelial, stromal, and endothelial cells. Together with their scalable derivation from somatic cells, these features highlight drNKs as a renewable and clinically viable source for regenerative strategies.

Transcriptomic profiling revealed enrichment of genes involved in tissue remodeling (*GREM2*, *FAM3C*), vesicular trafficking (*TXLNA*), and intercellular signaling (*CD40LG*, *CXCL16*), together with chemokine receptors (*CCR1*, *CCR2*, *CCR5*, *CCR6, CXCR3*). Enrichment analyses highlighted cytokine signaling, ECM-receptor interactions, PI3K-Akt, and lysosomal pathways. Consistently, cytokine arrays and ELISA showed that drNKs secreted markedly higher levels of CCL3, CCL4, and CCL5 compared with pNK-CM, along with CXCL12, GM-CSF, ICAM1, IFNγ, IL16, MIF, and TNFα, while IL2 was reduced. Although pNK-CM contained some CCR5 ligands, their levels were significantly lower. These differences were validated at both the transcript and protein levels and account for the limited regenerative efficacy of pNK-CM. Collectively, this establishes drNKs as a phenotypically distinct subset enriched in CCL3/4/5-CCR5 signaling, with a secretome biased toward repair rather than cytolysis.

Mechanistically, CCR5 signaling emerged as the dominant driver of drNK-CM activity. drNK-derived chemokines stimulated fibroblast, keratinocyte, and endothelial proliferation, migration, collagen synthesis, and angiogenesis, effects that were largely suppressed by maraviroc (MVC). Neutralization assays demonstrated that CCL5 exerted the strongest effect, while CCL3 and CCL4 contributed cooperatively, with combined blockade approximating MVC suppression. These findings reveal a synergistic mechanism in which multiple CCR5 ligands act redundantly but also reinforce one another to sustain robust regenerative signaling. MVC did not fully abolish activity, however, suggesting the presence of additional CCR5-independent mediators within the drNK secretome.

Angiogenesis was a central outcome of drNK-CM activity. *In vitro*, drNK-CM enhanced HUVEC proliferation, migration, and tube formation more effectively than pNK-CM, with suppression by MVC or CCL3/4/5 blockade confirming CCR5 dependence. Importantly, drNK-CM activity extended beyond CCR5 ligands: *VEGF* and *ANGPT1/2* were strongly upregulated, supporting both endothelial sprouting and vascular stabilization 41-43. *In vivo*, drNK-CM significantly increased CD31^+^ endothelial area and recruitment of CCR5^+^ stromal and immune cells, all of which were suppressed by MVC. Compared with recombinant VEGF, drNK-CM not only produced stronger angiogenic responses but also yielded more mature vascular networks, highlighting the therapeutic advantage of multifactorial secretomes over single-factor therapies 44-47.

These vascular effects were accompanied by immunoregulatory changes detected within wound sites. drNK-CM increased *IL11*, *IL1RA*, and *IL4* while reducing *IL1B*, indicating a shift toward an anti-inflammatory milieu conducive to tissue repair. In parallel, upregulation of *ANGPT1*, *ANGPT2*, *CD105*, and *CD31* supported enhanced vessel maturation. At the protein level, increased VEGF together with phosphorylation of Akt and ERK reflected activation of canonical pro-angiogenic and pro-survival pathways. Together, these findings demonstrate that drNK-CM simultaneously engages angiogenic and immunoregulatory programs in the wound microenvironment, thereby amplifying its regenerative efficacy.

ECM remodeling represented another major contribution of drNK-CM. *COL1A1* and *COL3A1* were strongly upregulated, together with *MMP1*, *MMP2*, *MMP3*, and *MMP9*. Protein analyses confirmed increased type I collagen, VEGF, MMP9, and both TGFβ1 and TGFβ3. While TGFβ1 promotes fibroblast activation and collagen deposition, TGFβ3 is associated with scar reduction and regenerative healing 48, 49. Their combined induction, along with enhanced MMP activity, suggests that drNK-CM establishes a balanced ECM environment that supports constructive deposition while limiting fibrosis. Histological analyses (H&E and Masson's trichrome) further confirmed enhanced re-epithelialization, dermal reconstruction, and collagen organization in drNK-CM-treated wounds. These effects were consistently stronger with drNK-CM than with pNK-CM, underscoring the impact of reprogramming.

Functionally, drNK-CM accelerated closure more efficiently than pNK-CM, with concentrated drNK-CCM producing near-complete repair. MVC or combined CCL3/4/5 neutralization delayed closure, confirming CCR5 dependence, whereas CCR1 or CCR3 inhibition had minimal effects. From a translational perspective, drNK-CM offers distinct advantages over live-cell or single-factor therapies. Unlike viable NKs, which face challenges of immune compatibility, cryopreservation, and GMP-scale expansion, drNK-CM is non-cytotoxic, easily standardized, and scalable. Unlike recombinant VEGF, which primarily targets angiogenesis, drNK-CM activates epithelial, stromal, vascular, and immune compartments in our assays, providing coordinated and multifactorial support for repair. These outcomes reflect the combined actions of CCR5 ligands, growth factors, and cytokines within the drNK secretome, offering robustness and redundancy while potentially reducing the risks associated with single-factor therapies 45-47.

Limitations should be acknowledged. Our *in vivo* analyses were restricted to acute murine wounds, and future studies in chronic, ischemic, and large-animal models are necessary. MVC did not completely block regenerative effects, underscoring the contribution of CCR5-independent factors that remain to be characterized. Finally, while Sendai virus reprogramming was effective, integration-free platforms such as episomal plasmids or synthetic mRNA would further improve biosafety for clinical application.

In conclusion, drNK-CM represents a novel regenerative secretome that orchestrates epithelial, stromal, vascular, and immune repair through CCR5-centered, multi-ligand synergy. Its broad efficacy derives from integrated, systems-level interactions rather than single-factor activity, distinguishing it from existing monotherapies. These findings establish drNK-CM as a clinically relevant, scalable, and versatile platform for wound healing and potentially broader regenerative applications.

## Supplementary Material

Supplementary figures and tables.
