# Supplementary material for: Cell-free secretome of CD56brightCD16bright directly reprogrammed NK cells enhances wound healing via CCL3/4/5-CCR5 signaling
Source: Theranostics. 2026 Jan 1;16(2):952–69. doi: 10.7150/thno.120219 (PMC12675002; doi:10.7150/thno.120219)
Supplement: Supplementary file 1 — Supplementary figures and tables. [file thnov16p0952s1.pdf]

Supplemental information includes 10 figures and 2 tables.

Cell-free secretome of CD56^{bright}CD16^{bright} directly reprogrammed NK cells enhances wound healing *via* CCL3/4/5-CCR5 signaling

Jae Yun Kim[1], Han-Seop Kim[1], Binna Seol[1], Ji Eun Choi[1, 2], Ji-Young Lee[1], and Yee Sook Cho [1, 2,*]

[1]Stem Cell Research Laboratory, Immunotherapy Research Center, Korea Research Institute of Bioscience and Biotechnology (KRIBB), 125 Gwahak-ro, Yuseong-gu, Daejeon 34141, Republic of Korea

[2]Department of Bioscience, KRIBB School, University of Science & Technology, 113 Gwahak-ro, Yuseong-gu, Daejeon 34113, Republic of Korea

[*]**Corresponding author**:

Yee Sook Cho, Ph.D. (E-mail: june@kribb.re.kr)

Contents of Supplemental Information:

This file contains supplemental data supporting the main manuscript. The following supplemental items are included:

Figure S1: Phenotypic characterization of directly reprogrammed NKs (drNKs) over extended culture.

Figure S2: Functional cytotoxicity of drNKs.

Figure S3: Comparative gene expression profiling between pNKs and drNKs.

Figure S4: Dose-dependent proliferative effects of drNK-conditioned medium (drNK-CM) on HEKs, HDFs, and HUVECs.

Figure S5: Effects of CCR1 and CCR3 blockade on drNK-CM-induced Type I collagen and chemokine receptor expression in HEKs and HDFs.

Figure S6: Dose-dependent effects of CCR1, CCR3, and CCR5 antagonists on the cell viability in HEKs, HDFs, and HUVECs.

Figure S7:Comparison of drNK-CM with recombinant VEGF (rVEGF) in angiogenesis assays.

Figure S8: Effects of CCR1 and CCR3 antagonists on drNK-CM-mediated wound healing in an excisional wound model.

Figure S9: Effects of CCL3/4/5 neuralization on the wound healing efficacy of drNK-CM

Figure S10: Comparison of drNK-CM and rVEGF in *in vivo* wound healing.

Table S1: Primer sequences used for qRT-PCR analysis.

Table S2: List of antibodies used for Western blot and immunohistochemistry.

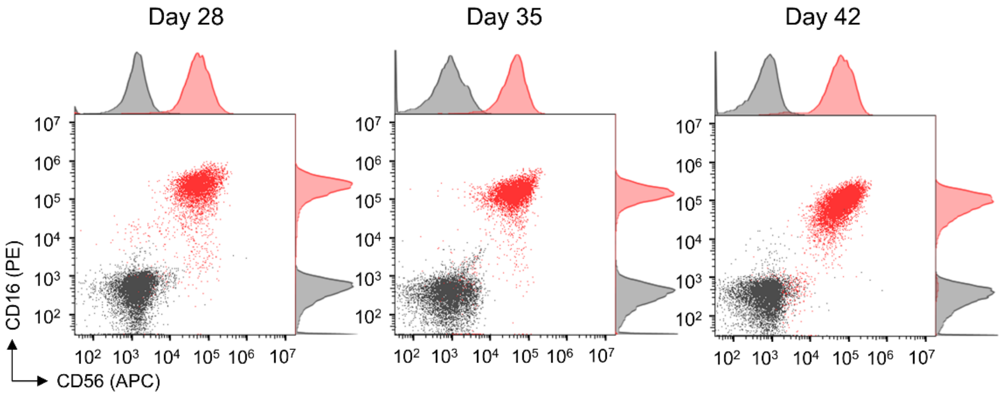

Figure S1. Phenotypic characterization of directly reprogrammed NKs (drNKs) during extended culture. Representative flow cytometry plots showing CD56 and CD16 expression at days 28, 35, and 42 of culture. Each plot represents one experiment; results were consistent across three independent biological replicates.

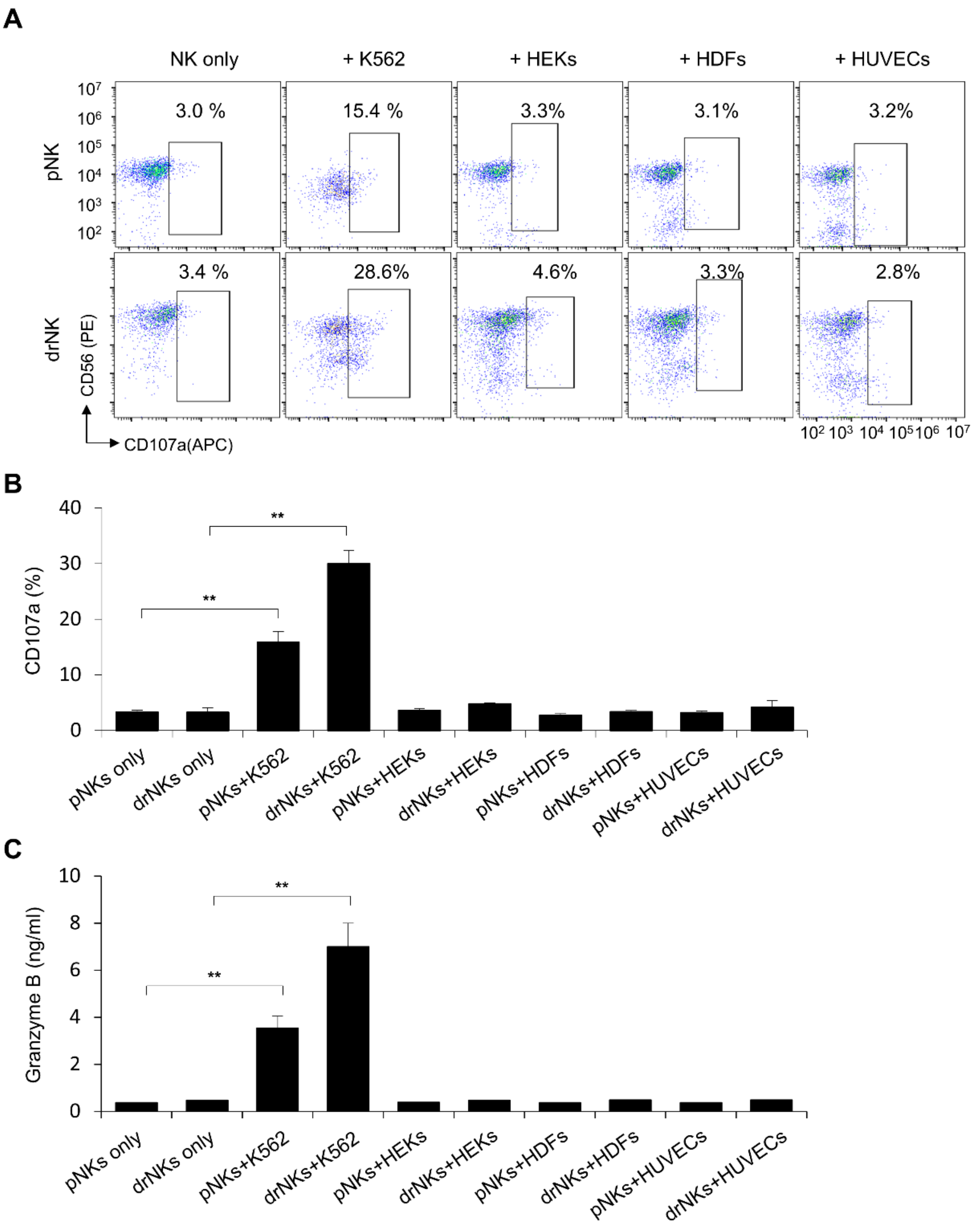

Figure S2. Functional cytotoxicity of drNKs. (A) Representative flow cytometry plots of CD107a degranulation in peripheral blood NKs (pNKs) and drNKs after 4 h co-culture with K562, HEKs, HDFs, or HUVECs at an effector-to-target ratio (E:T) of 1:1. **(B)** Quantification of CD107a^{+} NK cells under the conditions shown in (A). Data represent mean ± SEM (n = 3). **(C)** Extracellular granzyme B levels measured by ELISA after 8 h co-culture at E:T = 1:1 with

the indicated targets. Data represent mean ± SEM (n = 3). Statistical significance was determined using an unpaired two-tailed Student's t-test. *$p < 0.05$, **$p < 0.01$ compared with indicated control groups.

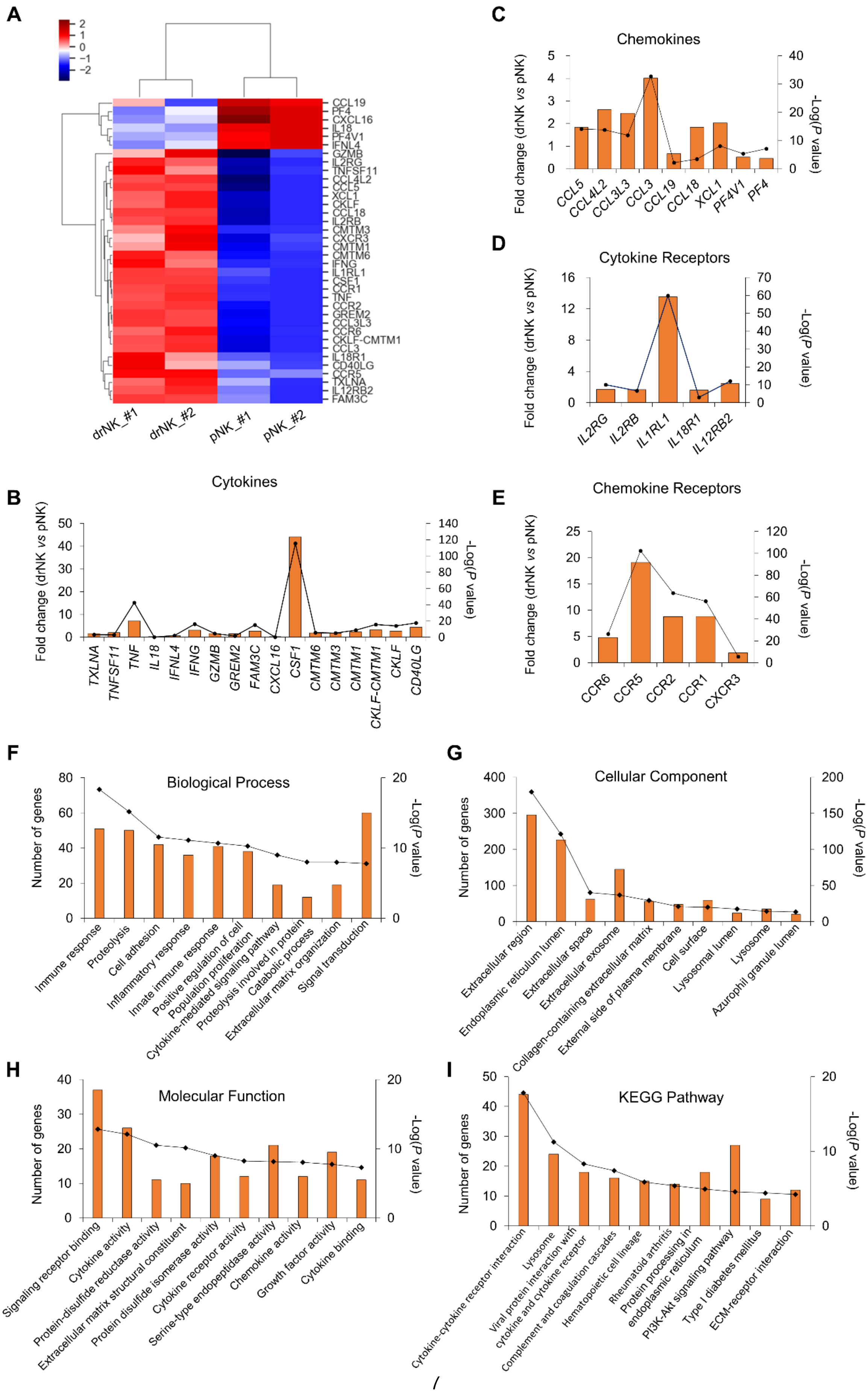

A
2
1
0
−1
−2
CCL19
PF4
CXCL16
IL18
PF4V1
IFNL4
GZMB
IL2RG
TNFSF11
CCL4L2
CCL5
XCL1
CKLF
CCL18
IL2RB
CMTM3
CXCR3
CMTM1
CMTM6
IFNG
IL1RL1
CSF1
CCR1
TNF
CCR2
GREM2
CCL3L3
CCR6
CKLF-CMTM1
CCL3
IL18R1
CD40LG
CCR5
TXLNA
IL12RB2
FAM3C
drNK_#1
drNK_#2
pNK_#1
pNK_#2
B
Cytokines
Fold change (drNK vs pNK)
-Log(P value)
TXLNA
TNFSF11
TNF
IL18
IFNL4
IFNG
GZMB
GREM2
FAM3C
CXCL16
CSF1
CMTM6
CMTM3
CMTM1
CKLF-CMTM1
CKLF
CD40LG
C
Chemokines
Fold change (drNK vs pNK)
-Log(P value)
CCL5
CCL4L2
CCL3L3
CCL3
CCL19
CCL18
XCL1
PF4V1
PF4
D
Cytokine Receptors
Fold change (drNK vs pNK)
-Log(P value)
IL2RG
IL2RB
IL1RL1
IL18R1
IL12RB2
E
Chemokine Receptors
Fold change (drNK vs pNK)
-Log(P value)
CCR6
CCR5
CCR2
CCR1
CXCR3
F
Biological Process
Number of genes
-Log(P value)
Immune response
Proteolysis
Cell adhesion
Inflammatory response
Innate immune response
Positive regulation of cell
Population proliferation
Cytokine-mediated signaling pathway
Proteolysis involved in protein
Catabolic process
Extracellular matrix organization
Signal transduction
G
Cellular Component
Number of genes
-Log(P value)
Extracellular region
Endoplasmic reticulum lumen
Extracellular space
Extracellular exosome
Collagen-containing extracellular matrix
External side of plasma membrane
Cell surface
Lysosomal lumen
Lysosome
Azurophil granule lumen
H
Molecular Function
Number of genes
-Log(P value)
Signaling receptor binding
Cytokine activity
Protein-disulfide reductase activity
Extracellular matrix structural constituent
Protein disulfide isomerase activity
Cytokine receptor activity
Serine-type endopeptidase activity
Chemokine activity
Growth factor activity
Cytokine binding
I
KEGG Pathway
Number of genes
-Log(P value)
Cytokine-cytokine receptor interaction
Lysosome
Viral protein interaction with
cytokine and cytokine receptor
Complement and coagulation cascades
Hematopoietic cell lineage
Rheumatoid arthritis
Protein processing in
endoplasmic reticulum
PI3K-Akt signaling pathway
Type I diabetes mellitus
ECM-receptor interaction

/

Figure S3. Comparative gene expression profiling of pNKs and drNKs. (A) Heatmap showing differential transcriptional profiles of pNKs versus drNKs. **(B-E)** Fold-change comparisons (drNKs versus pNKs) of genes encoding cytokines (*TXLNA*, *TNFSF11*, *TNF*, *IL18*, *IFNL4*, *IFNG*, *GZMB*, *GREM2*, *FAS3C*, *CXCL16*, *CSF1*, *CMTM6*, *CMTM3*, *CMTM1*, *CKLF*, *CD40LG*) (B), chemokines (*CCL5*, *CCL4L2*, *CCL3L3*, *CCL3*, *CCL19*, *CCL18*, *XCL1*, *PF4V1*, *PF4*) (C), cytokine receptors (*IL2RG*, *IL2RB*, *IL1RL1*, *IL18R1*, *IL12RB2*) (D), and chemokine receptors (*CCR6*, *CCR2*, *CCR1*, *CXCR3*) (E). **(F-H)** Gene Ontology (GO) enrichment analyses of differentially expressed secretome-related genes (n = 450, FDR < 0.05), categorized by biological processes (F), cellular components (G), and molecular functions (H). **(I)** Kyoto Encyclopedia of Genes and Genomes (KEGG) pathway enrichment analysis of the same gene set. Functional annotation of differentially expressed genes (DEGs) was performed using the DAVID online server.

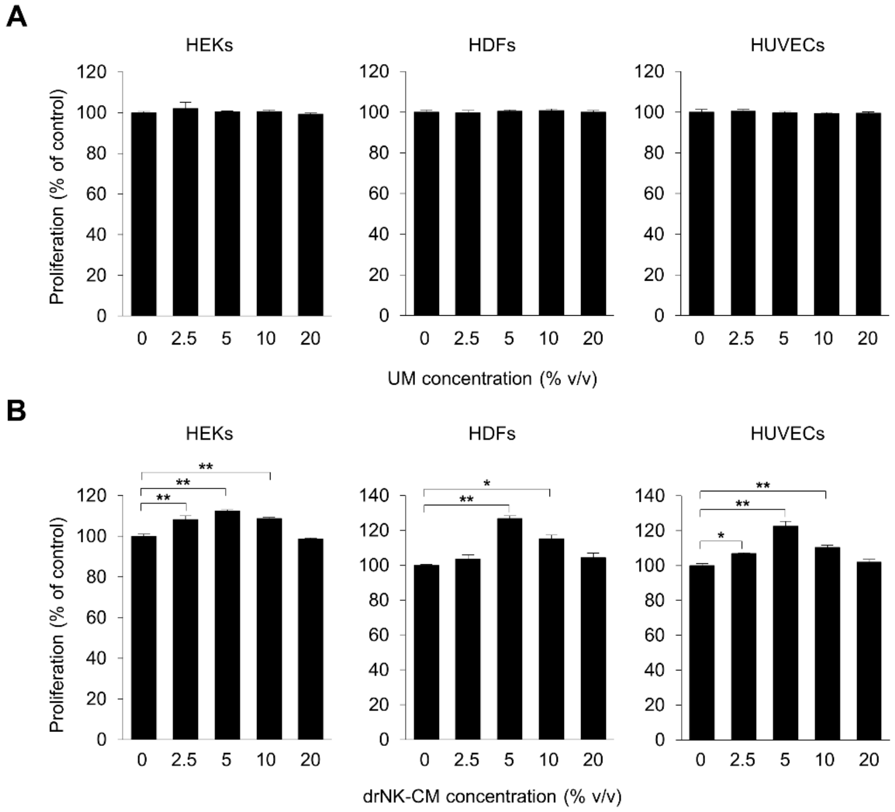

Figure S4. Dose-dependent proliferative effects of drNK-conditioned medium (drNK-CM) on HEKs, HDFs, and HUVECs. (A-B) Quantification of cell proliferation in HEKs, HDFs, and HUVECs cultured in unconditioned medium (UM) or medium supplemented with increasing concentrations of drNK-CM (0, 2.5, 5, 10, 20%) for 48 h. Cell proliferation was assessed using the MTT assay. Data represent mean ± SEM (n = 3). One-way ANOVA with Tukey's post-hoc test; $*p < 0.05$, $**p < 0.01$ compared with untreated control groups.

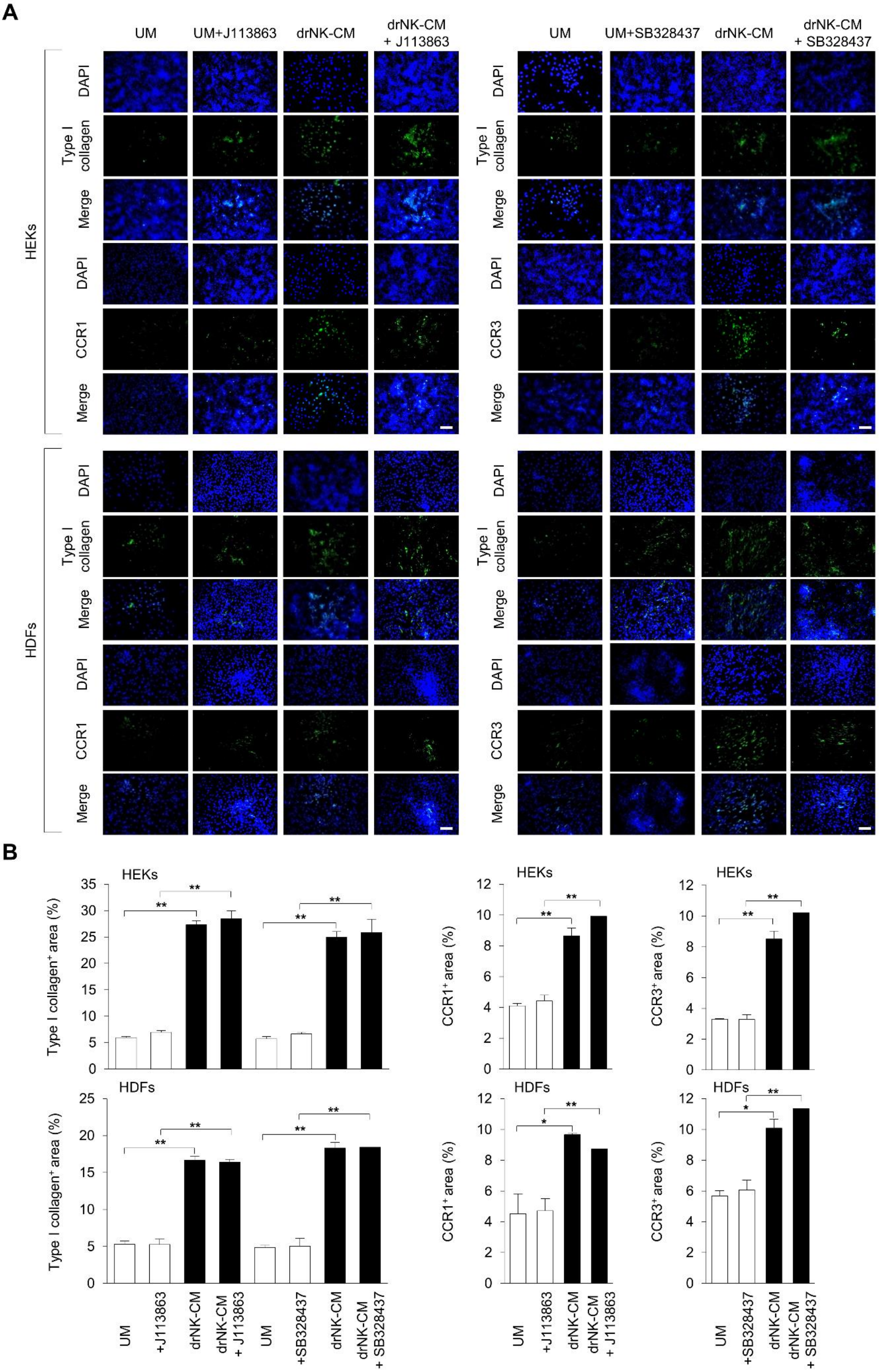
A
UM
UM+J113863
drNK-CM
drNK-CM + J113863
UM
UM+SB328437
drNK-CM
drNK-CM + SB328437
HEKs
HDFs
DAPI
Type I collagen
Merge
CCR1
CCR3
B
HEKs
HDFs
Type I collagen⁺ area (%)
CCR1⁺ area (%)
CCR3⁺ area (%)
**
*
UM
+J113863
drNK-CM
drNK-CM + J113863
+SB328437
drNK-CM + SB328437

Figure S5. Effects of CCR1 and CCR3 blockade on drNK-CM-induced Type I collagen and chemokine receptor expression in HEKs and HDFs. (**A**) Representative immunocytochemistry images showing collagen type I, CCR1, and CCR3 expression in HEKs and HDFs treated with UM or drNK-CM, in the presence or absence of CCR1 antagonist J113863 or CCR3 antagonist SB328437. Scale bar = 100 μm. (**B**) Quantitative analysis of Type I collagen^{+}, CCR1^{+}, and CCR3^{+} area from (A). Data represent mean ± SEM (n = 3). One-way ANOVA with Tukey's post-hoc test; *$p < 0.05$, **$p < 0.01$ compared with indicated control groups.

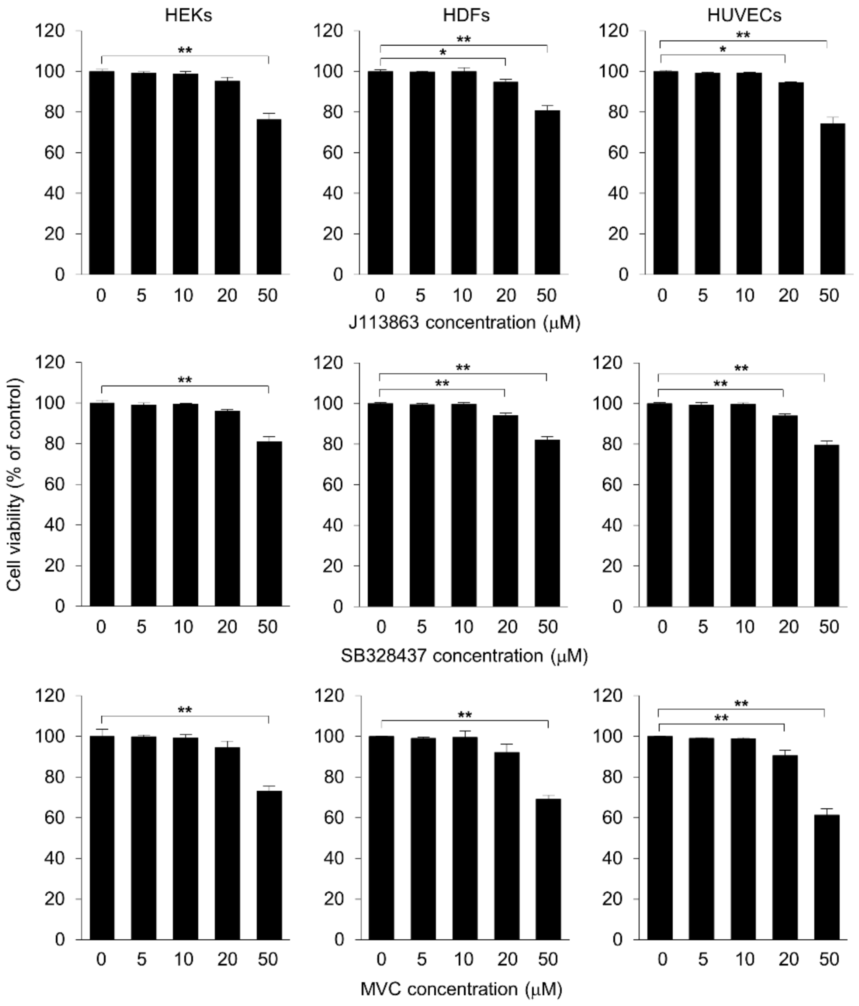

Figure S6. Dose-dependent effects of CCR1, CCR3, and CCR5 antagonists on cell viability in HEKs, HDFs, and HUVECs. Cells were treated with increasing concentrations (0, 5, 10, 20, 50 μM) of J113863, SB328437, or CCR5 antagonist maraviroc (MVC). Cell viability was assessed after 48 h using the MTT assay. Data represent mean ± SEM (n = 3). One-way ANOVA with Tukey's post-hoc test; *$p < 0.05$, **$p < 0.01$ compared with untreated control groups.

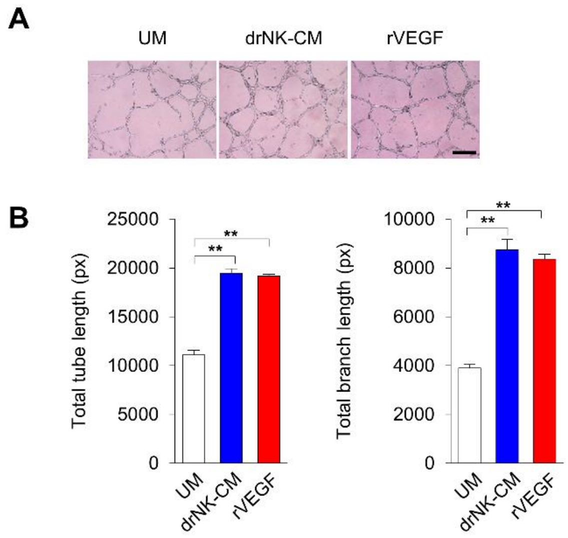

Figure S7. Comparison of drNK-CM with recombinant VEGF (rVEGF) in angiogenesis assays. (A) Representative images of HUVEC tube formation under UM, 5% drNK-CM, or rVEGF (10 ng/mL). Scale bar = 100 µm. **(B)** Quantification of total tube length (left) and branch points (right) per field. Data are mean ± SEM (n = 3). Statistical significance was determined using a two-tailed Student's t-test. $*p < 0.05$, $**p < 0.01$ compared with UM.

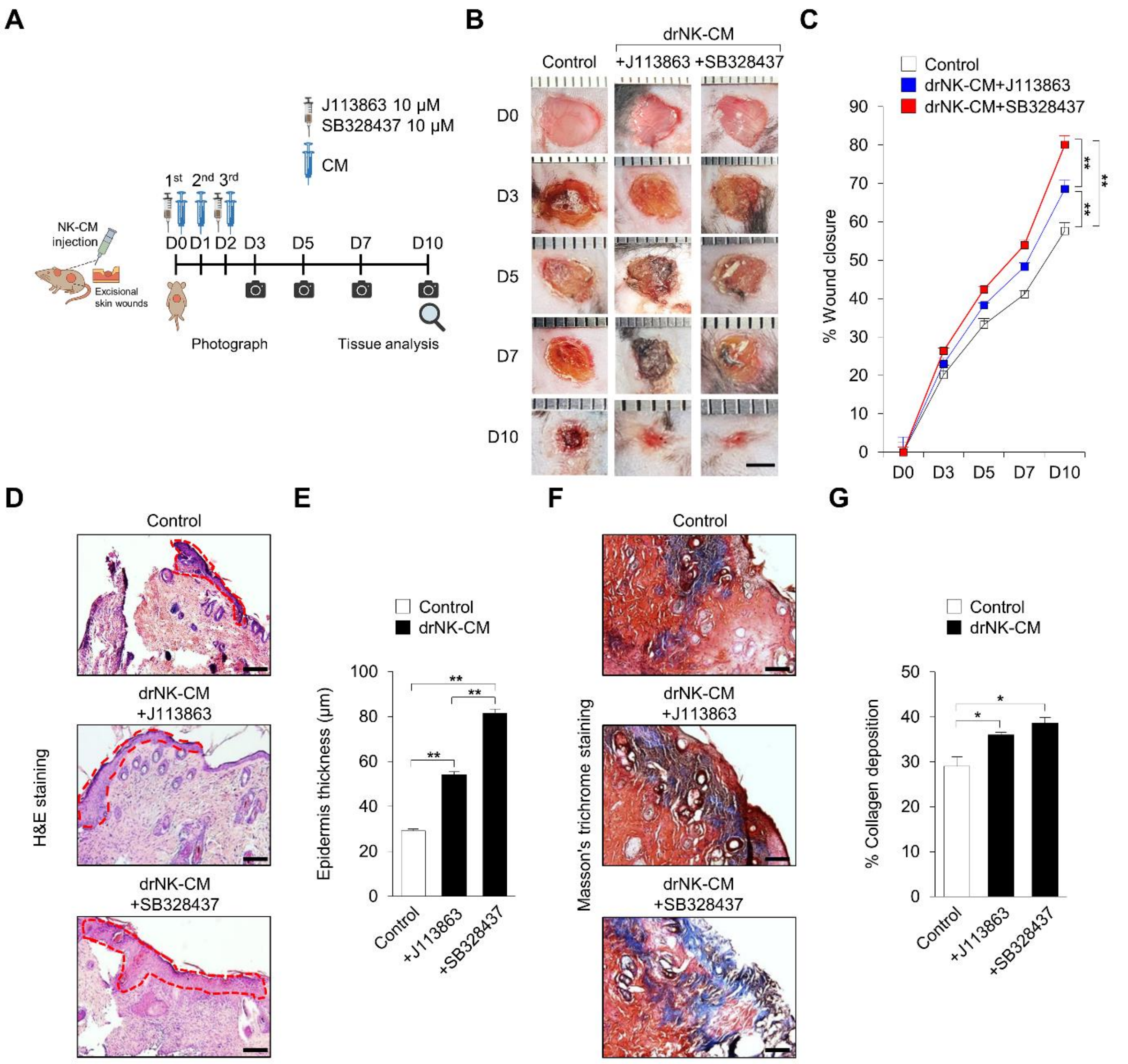

Figure S8. Effects of CCR1 and CCR3 antagonists on drNK-CM-mediated wound healing in an excisional wound model. (A) Experimental timeline of excisional wounding, drNK-CM application, and CCR1/CCR3 antagonist administration. **(B)** Representative macroscopic images of wound closure with or without J113863 or SB328437. **(C)** Quantification of wound closure rate in each group from (B). Data represent mean ± SEM (n = 3). **(D)** Hematoxylin and eosin (H&E) staining showing epithelial regeneration. **(E)** Quantification of epidermal thickness from (D). **(F)** Masson's trichrome staining showing collagen deposition. Scale bar = 100 μm. **(G)** Quantification of collagen deposition from (F). Data represent mean ± SEM (n =

4 mice per group). Scale bar = 100 μm. One-way ANOVA with Tukey's post-hoc test; $*p < 0.05$, $**p < 0.01$ compared with corresponding control groups. Randomization and blinded assessment were applied.

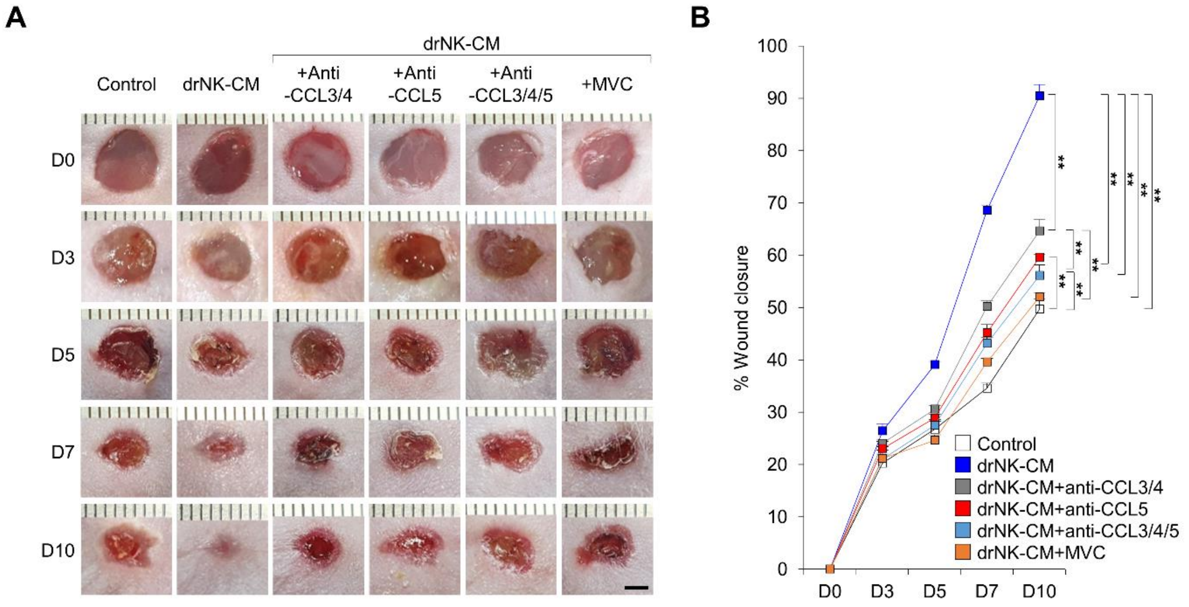

Figure S9. Effects of CCL3/4/5 neuralization on the wound healing efficacy of drNK-CM. (A) Representative wound images on days 0, 3, 5, 7, and 10 following treatment with control or drNK-CM, in the presence or absence of anti-CCL3/4, anti-CCL5, or anti-3/4/5 antibodies (10 ng/mL), or MVC (10 μM). Scale bar = 100 μm. **(B)** Quantification of wound closure over time, expressed as % of initial wound area. Data represent mean ± SEM (n = 4 mice per group. One-way ANOVA with Tukey's post-hoc test; *$p < 0.05$, **$p < 0.01$ compared with corresponding control groups. Randomization and blinded assessment were applied.

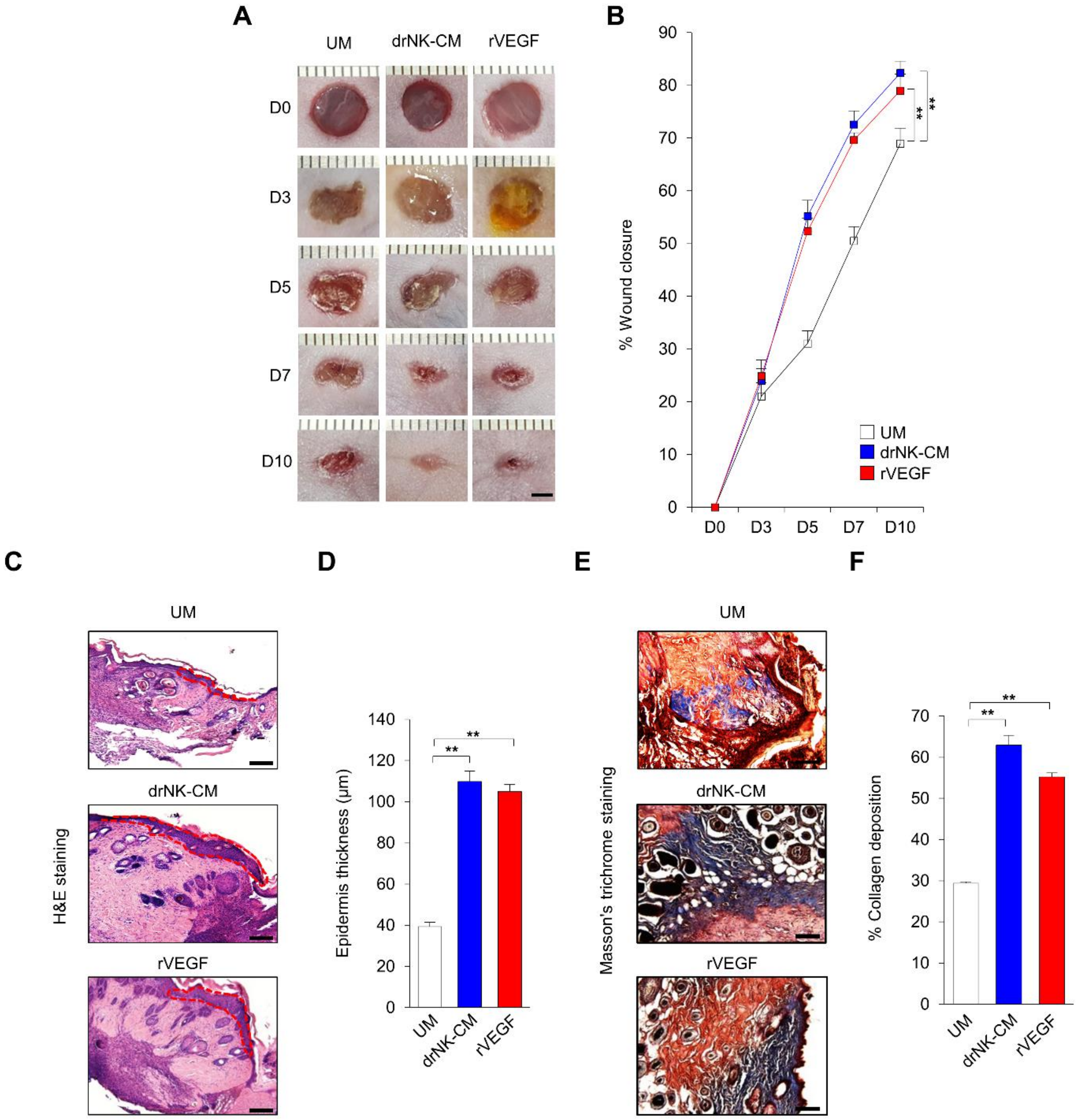

Figure S10. Comparison of drNK-CM and rVEGF in *in vivo* wound healing. **(A)** Representative images of excisional wounds on days 0, 3, 5, 7, and 10 following topical treatment with PBS (control), UM, 5% drNK-CM, or rVEGF (25 μg/mL). **(B)** Wound closure curves over time (% relative to day 0). **(C)** H&E staining of wound sections at day 7 showing epithelial regeneration. Scale bar = 100 μm. **(D)** Quantification of epidermal thickness from (C). Data represent mean ± SEM (n = 3). **(E)** Masson's trichrome staining on day 10 showing collagen deposition. **(F)** Quantification of collagen^{+} area from (E). Scale bar = 100 μm. Data

represent mean ± SEM (n = 4 mice per group). Statistical significance was determined using a two-tailed Student's t-test. *$p < 0.05$, **$p < 0.01$ compared with corresponding control groups.

Table S1. List of primers used in this study.

Gene	Sequence (5' → 3')	
CCR1	*Forward*	CCT GCT GAC GAT TGA CAG GTA
	Reverse	TCT CGT AGG CTT TCG TGA GGA
CCR3	*Forward*	TGG CAT GTG TAA GCT CCT CTC
	Reverse	CCT GTC GAT TGT CAG CAG GAT TA
CCR5	*Forward*	TTG CCA AAC GCT TCT GCA AAT
	Reverse	AGT GGA TCG GGT GTA AAC TGA
COL1A1	*Forward*	GAG GGC CAA GAC GAA GAC ATC
	Reverse	CAG ATC ACG TCA TCG CAC AAC
COL3A1	*Forward*	TTG AAG GAG GAT GTT CCC ATC T
	Reverse	ACA GAC ACA TAT TTG GCA TGG TT
MMP-1	*Forward*	CTC TGG AGT AAT GTC ACA CCT CT
	Reverse	TGT TGG TCC ACC TTT CAT CTT C
MMP-2	*Forward*	CCC ACT GCG GTT TTC TCG AAT
	Reverse	CAA AGG GGT ATC CAT CGC CAT
MMP-3	*Forward*	CTG GAC TCC GAC ACT CTG GA
	Reverse	CAG GAA AGG TTC TGA AGT GAC C
MMP-9	*Forward*	GGG ACG CAG ACA TCG TCA TC
	Reverse	TCG TCA TCG TCG AAA TGG GC
TIMP-1	*Forward*	AGA GTG TCT GCG GAT ACT TCC
	Reverse	CCA ACA GTG TAG GTC TTG GTG
TGFβ-1	*Forward*	CAA TTC CTG GCG ATA CCT CAG
	Reverse	GCA CAA CTC CGG TGA CAT CAA
TGFβ-3	*Forward*	AAC GGT GAT GAC CCA CGT C
	Reverse	CCG ACT CGG TGT TTT CCT GG
ANGPT1	*Forward*	AGC GCC GAA GTC CAG AAA AC
	Reverse	TAC TCT CAC GAC AGT TGC CAT
ANGPT2	*Forward*	AAC TTT CGG AAG AGC ATG GAC
	Reverse	CGA GTC ATC GTA TTC GAG CGG
CCL3	*Forward*	AGT TCT CTG CAT CAC TTG CTG
	Reverse	CGG CTT CGC TTG GTT AGG AA
CCL4	*Forward*	CTG TGC TGA TCC CAG TGA ATC
	Reverse	TCA GTT CAG TTC CAG GTC ATA CA
CCL5	*Forward*	CCA GCA GTC GTC TTT GTC AC
	Reverse	CTC TGG GTT GGC ACA CAC TT

VEGF	*Forward*	GCT CAG AGC GGA GAA AGC AT
	Reverse	TCA GTC TTT CCT GGT GAG AGA T
VEGFR2	*Forward*	AGC GAT GGC CTC TTC TGT AA
	Reverse	ACA CGA CTC CAT GTT GGT CA
GAPDH	*Forward*	GAA GGT GAA GGT CGG AGT C
	Reverse	GAA GAT GGT GAT GGG ATT TC
β-actin	*Forward*	CAC CAT TGG CAA TGA GCG GTT C
	Reverse	AGG TCT TTG CGG ATG TCC ACG T
mCCL5	*Forward*	GCT GCT TTG CCT ACC TCT CC
	Reverse	TCG AGT GAC AAA CAC GAC TGC
mCOL1A1	*Forward*	AGA CAG TGA TTG AAT ACA AAA CCA
	Reverse	GGA GTT TAC AGG AAG CAG ACA
mCOL3A1	*Forward*	CTG TAA CAT GGA AAC TGG GGA AA
	Reverse	CCA TAG CTG AAC TGA AAA CCA CC
mVEGF	*Forward*	CTC CGC TCT GAA CAA GGC T
	Reverse	TCC TGT TGC TGT GCT CTG CT
mANGPT1	*Forward*	ATC CCG ACT TGA AAT ACA ACT GC
	Reverse	CTG GAT GAT GAA TGT CTG ACG AG
mANGPT2	*Forward*	GGA CAG TCA TCC AAC ACC GAG
	Reverse	GAC TCT TCA CCA GCG AGG TAG
mCD105	*Forward*	AGG GGT GAG GTG ACG TTT AC
	Reverse	GTG CCA TTT TGC TTG GAT GC
mCD31	*Forward*	ACC GGG TGC TGT TCT ATA AGG
	Reverse	TCA CCT CGT ACT CAA TCG TGG
mIL11	*Forward*	TGT TCT CCT AAC CCG ATC CCT
	Reverse	CAG GAA GCT GCA AAG ATC CCA
mIL1β	*Forward*	GAA ATG CCA CCT TTT GAC AGT G
	Reverse	TAC CAG TTG GGG AAC TCT GC
mIL1RA	*Forward*	TAG ACA TGG TGC CTA TTG ACC T
	Reverse	TCG TGA CTA TAA GGG GCT CTT C
mIL4	*Forward*	GGT CTC AAC CCC CAG CTA GT
	Reverse	GCC GAT GAT CTC TCT CAA GTG AT
mGAPDH	*Forward*	AGG TCG GTG TGA ACG GAT TTG
	Reverse	TGT AGA CCA TGT AGT TGA GGT CA

Table S2. List of antibodies used in this study.

Antigen	Species	Applications	References	Source
CCL3	Rabbit	WB (1:1000)	ab259372	Abcam
CCL3	Goat	WB (1:1000)	AF-270-NA	R&D Systems
CCL4	Rabbit	WB (1:1000)	ab45690	Abcam
CCL4	Goat	WB (1:1000)	AF-271-NA	R&D Systems
CCL5	Goat	WB (1:1000)	AF-278-SP	R&D Systems
GAPDH	Mouse	WB (1:2000)	sc-47724	Santa Cruz
GAPDH	Mouse	WB (1:2000)	sc-166545	Santa Cruz
COL1A1	Rabbit	WB (1:1000)<br>IF/IHC (1:400)	ab34710	Abcam
COL1A1	Rabbit	WB (1:1000)<br>IF/IHC (1:400)	PA5-29569	Invitrogen
CCR1	Rabbit	IF/IHC (1:200)	NB100-56334	Novus Biologicals
CCR3	Rabbit	IF/IHC (1:200)	NBP1-77065	Novus Biologicals
CCR5	Rabbit	IF/IHC (1:200)	NBP2-31374	Novus Biologicals
MMP9	Mouse	WB (1:1000)	sc-13520	Santa Cruz
TGFβ-1	Rabbit	WB (1:1000)	ab92486	Abcam
VEGF	Mouse	WB (1:1000)	MA5-13182	Invitrogen
CD31<br>(PECAM-1)	Rabbit	IF/IHC (1:200)	orb317590	Biorbyt
CD31	Rabbit	IF/IHC (1:200)	BS-0468R	Bioss
Akt	Rabbit	WB (1:1000)	#9272	CST
Akt	Rabbit	WB (1:1000)	#4685	CST
p-Akt	Rabbit	WB (1:1000)	#9271	CST
p-Akt	Rabbit	WB (1:1000)	#9275	CST
Erk	Rabbit	WB (1:1000)	#9102	CST
p-Erk	Rabbit	WB (1:1000)	#4370	CST
β-actin	Mouse	WB (1:2000)	sc-47778	Santa Cruz
Rabbit IgG-HRP	Mouse	WB (1:200)	sc-2357	Santa Cruz
Rabbit IgG-HRP	Goat	WB (1:500)	sc-2004	Santa Cruz
Mouse IgG-HRP	Goat	WB (1:500)	sc-2005	Santa Cruz
Mouse IgM-HRP	Goat	WB (1:500)	sc-2973	Santa Cruz
Goat IgG-HRP	Mouse	WB (1:500)	sc-2354	Santa Cruz
Alexa Flour 488 donkey<br>anti-mouse IgG	Donkey	IF/IHC (1:200)	A21202	Invitrogen

Alexa Flour 488 goat anti-mouse IgG	Goat	IF/IHC (1:200)	A11001	Invitrogen
Alexa Flour 594 goat anti-mouse IgG2a	Goat	IF/IHC (1:200)	A21135	Invitrogen
Cy3 goat anti-mouse IgG	Goat	IF/IHC (1:200)	A10521	Invitrogen
Alexa Flour 488 donkey anti-rabbit IgG	Donkey	IF/IHC (1:200)	A21206	Invitrogen
Alexa Flour 594 donkey anti-rabbit IgG	Donkey	IF/IHC (1:200)	A21207	Invitrogen
Alexa Flour 594 chicken anti-rabbit IgG	A21442	IF/IHC (1:400)	A21442	Invitrogen
